# A Semi-Dominant Mutation in OsCESA9 Improves Salt Tolerance and Favors Field Straw Decay Traits by Altering Cell Wall Properties in Rice

**DOI:** 10.1186/s12284-021-00457-0

**Published:** 2021-02-17

**Authors:** Yafeng Ye, Shuoxun Wang, Kun Wu, Yan Ren, Hongrui Jiang, Jianfeng Chen, Liangzhi Tao, Xiangdong Fu, Binmei Liu, Yuejin Wu

**Affiliations:** 1 Key Laboratory of High Magnetic Field and Ion Beam Physical Biology, Hefei Institutes of Physical Science, Chinese Academy of Sciences, Hefei, 230031 China; 2 Anhui Province Key Laboratory of Environmental Toxicology and Pollution Control Technology, Hefei Institutes of Physical Science, Chinese Academy of Sciences, Hefei, 230031 Anhui China; 3 State Key Laboratory of Plant Cell and Chromosome Engineering, Institute of Genetics and Developmental Biology, The Innovative Academy of Seed Design, Chinese Academy of Sciences, Beijing, 100101 China

**Keywords:** Secondary cell wall (SCW), Rice, Cellulose synthesis, Salt tolerance, Straw process

## Abstract

**Background:**

Cellulose synthase (CESA) mutants have potential use in straw processing due to their lower cellulose content, but almost all of the mutants exhibit defective phenotypes in plant growth and development. Balancing normal plant growth with reduced cellulose content remains a challenge, as cellulose content and normal plant growth are typically negatively correlated with one another.

**Result:**

Here, the rice (*Oryza sativa*) semi-dominant brittle culm (*sdbc*) mutant *Sdbc*1, which harbors a substitution (D387N) at the first conserved aspartic acid residue of OsCESA9, exhibits lower cellulose content and reduced secondary wall thickness as well as enhanced biomass enzymatic saccharification compared with the wild type (WT). Further experiments indicated that the OsCESA9^D387N^ mutation may compete with the wild-type OsCESA9 for interacting with OsCESA4 and OsCESA7, further forming non-functional or partially functional CSCs. The OsCESA9/OsCESA9^D387N^ heterozygous plants increase salt tolerance through scavenging and detoxification of ROS and indirectly affecting related gene expression. They also improve rice straw return to the field due to their brittle culms and lower cellulose content without any negative effects in grain yield and lodging.

**Conclusion:**

Hence, OsCESA9^D387N^ allele can improve rice salt tolerance and provide the prospect of the rice straw for biofuels and bioproducts due to its improved enzymatic saccharification.

**Supplementary Information:**

The online version contains supplementary material available at 10.1186/s12284-021-00457-0.

## Background

As one of the most important staple food crops, rice produces large quantities of lignocellulose biomass, which is a key component for the production of bio-energy and bio-based products. However, due to the lignocellulose recalcitrance of the secondary cell wall, the utilization of rice straw for bio-energy is very limited, and the processing of rice straw is very difficult (Himmel et al. 2007; Sathitsuksanoh et al. 2013). Biomass saccharification is an important indicator for enzymatic digestion to release soluble sugars, which is one of the major steps for conversion of lignocellulose to ethanol (Alam et al. 2019; Wang et al. 2016b). Cellulose is a large molecular polysaccharide composed of β-1, 4-D-glucan chains, which are synthesized individually and form microfibrils by intra- and intermolecular hydrogen bonds and Van der Waals forces (McFarlane et al. 2014). The cellulose microfibrils are the framework of the plant cell wall, and the hemi-celluloses and pectins or lignins fill in the gaps between the cellulose microfibrils and can cross-link cellulose microfibrils to form two different types of plant cell wall, namely, the primary cell wall and secondary cell wall (Hofte and Voxeur 2017). Hence, the cellulose content and crystallinity are key parameters negatively affecting biomass digestibility. Therefore, the genetic modification of cellulose is very useful for reducing lignocellulose recalcitrance for enhancing biomass saccharification and improving rice straw decomposition rates (Vermerris and Abril 2015).

In higher plants, cellulose synthesis is catalyzed by the plasma membrane-localized cellulose synthase complexes (CSCs) using uridine diphosphate (UDP)-glucose as a substrate (McFarlane et al. 2014). The functional CSC requires the cooperation of at least three distinct cellulose synthase (CESA) isoforms, which are encoded by different CESA genes (Taylor et al. 2003). Since the first higher plant CESA gene was cloned from cotton in 1996 (Pear et al. 1996), many CESA genes from different plant species have become publicly available (Joshi and Mansfield 2007). Plant CESA proteins are 986–1088 amino acids in length and share common domains and motifs. The zinc finger domains at the N-terminus, eight transmembrane domains (TMDs) and a central cytoplasmic domain with D,D,D,QXXRW motif between TMD2 and TMD3. The current model of cellulose synthesis predicts that the first two conserved aspartic acid residues coordinate UDP, the third plays a role in providing the catalytic base for the glucan extension, and the QXXRW residues function as a binding site for the terminal glucan residues of the chain (Somerville 2006). Mutations in these domains of CESA result in cellulose content decreases and defective growth phenotypes (McFarlane et al. 2014).

In rice, OsCESA4, OsCESA7 and OsCESA9 comprise the CSCs necessary for secondary cell wall cellulose synthesis (Tanaka et al. 2003). To date, numerous mutants in these three genes have been identified, all of these mutants show the brittle culm (*bc*) phenotype due to decreases in cellulose content (Kotake et al. 2011; Li et al. 2018; Li et al. 2017; Song et al. 2013; Tanaka et al. 2003; Wang et al. 2016a; Wang et al. 2012). Apart from the brittle culm phenotype, the OsCESA4 and OsCESA7 mutants exhibit abnormal plant growth (dwarfism, small leaves, withered leaf tips) (Wang et al. 2016a; Wang et al. 2012), only OsCESA9 conserved site mutations are able to maintain normal plant growth and grain production in plants. For example, the *bc13* mutant with one amino acid alteration (G101K) in the N-terminus of OsCESA9 shows normal plant growth and cadmium tolerance, despite a reduction in cellulose (Song et al. 2013). The *Osfc16* mutant with substitutions (W481C, P482S) at the P-CR conserved site in OsCESA9 shows slightly affected plant growth and maintains grain yields similar to its wild type, but not only that, it also enhances biomass saccharification due to much improved lignocellulose features such as cellulose the degree of polymerization (DP) & the crystallinity index (CrI) and nanocellulose fibers assembly (Li et al. 2017). *Bc6*, a semi-dominant brittle culm mutant harbors a substitution (R588G), which is located 203 amino acids before the catalytic motif QXXRW, and also shows a brittle culm without any pleiotropic phenotype such as dwarfism and withering (Kotake et al. 2011). Several OsCESA9 mutants have been identified, but only the *Bc6* is a semi-dominant brittle culm mutant (Kotake et al. 2011). To further interpret why only OsCESA9 mutants maintain normal plant growth and grain yield, more OsCESA9 mutants with conserved-site mutations need to be discovered, particularly semi-dominant mutants, in order to elucidate the function of the OsCESA9 conserved-site domain.

Soil salinity is one of the major stresses adversely affecting plant growth and crop productivity (Munns and Tester 2008). The identification of more salt-tolerant mutants is of vital importance for crop breeding in salt-affected fields. The plant cell wall plays a crucial role in development and in adaptation to abiotic and biotic stresses (Correa-Ferreira et al. 2019; Fang et al. 2018; Li et al. 2020; Palmeros-Suarez et al. 2017), and modulating secondary cell wall cellulose synthesis plays an important role in plant responses to salt stress (Chen et al. 2005). In this study, a novel semi-dominant brittle cum (*sdbc*) mutant, which harbors a substitution (D387N) at the first conserved aspartic acid residue of the CESA9 protein, was obtained by heavy ion beam treatment of the *japonica* cultivar Wuyunjing7. The WT/*sdbc1* heterozygotes show a mild brittle culm phenotype without any the appearance of pleiotropic phenotypes and also exhibit improved salt tolerance. Further evidence revealed that the mutation OsCESA9^D387N^ can compete with wild type OsCESA9 to form an abnormal CSC with OsCESA4 and OsCESA7.

## Methods

### Plant Materials and Growth Conditions

The *sdbc1* mutant was obtained from a *japonica* cultivar *Wuyunjing7* by heavy ion beam treatment (the energy of carbon ions is 80 MeV/nucleon, irradiation dose is 120Gy). The F_1_ plants and genetic analysis population were generated by the cross between *sdbc1* and its wild type (WT) *Wuyunjing7*. An F_2_ mapping population was generated from the cross between *sdbc1* and an *indica* cultivar, *93–11*. All rice plants used in this research were grown in the experimental fields at the Institute of Technical Biology and Agriculture Engineering, Hefei Institute of Physical Science, Chinese Academy of Sciences (Hefei, China) and Sanya (Hainan province, China) in the natural growing season.

### Measurement of Extension Force and Microscopy

Extension force of the second internodes and flag leaves of WT, *sdbc1* and F1 plants were determined according to (Ye et al. 2015). The maximum force required to break apart the internodes and leaves were considered as the mechanical strength of WT, *sdbc1* and F1 plants at heading stage.

For transmission electron microscopy, the second internodes of WT, *sdbc1* and F1 plants were cut with a razor and immediately post-fixed in 70% ethanol (V/V), 5% acetic acid (V/V), and 3.7% formaldehyde (V/V) mixture for at least 2 h. Samples were dried to the critical point, sputter-coated with gold, and observed with a scanning electron microscope (S570; Hitachi, Tokyo, Japan). The thickness of sclerenchyma cell wall was measured by Image J software.

### Phenotypic Evaluation

Several agronomic traits, including plant height, number of tillers per plant, number of grains per panicle, panicle length, setting rate, 1000-grain weight and lodging index were chosen for production evaluation. The plant height was measured from the ground surface to the tallest panicle. The number of tillers per plant was the number of effective tillers with corresponding panicle has 10 or more grains. The number of grains per panicle was the total number of grains per panicle. The panicle length was the distance from length top to the first internode top. The setting rate was scored as the number of full grains per panicle divided by the number of grains per panicle. The 1000-grain weight was calculated based on random number of grains (> 100) and then converted to 1000 grain weight. The plant lodging index was analyzed according to (Li et al. 2017). All traits were measured at full senescence. The data from fifty plants in each background in 2017 and 2018 was used for analysis. The independent sample *t*-test program SPSS 10.0 for Windows was used to compare the mean values.

### Cell Wall Composition Analysis and Saccharification Assays

The second internodes of WT, *sdbc1* and F1 plants at mature stage were used to prepare alcohol-insoluble residues (AIRs) of the cell walls. De-starched AIRs were produced as previously described (Zhou et al. 2009). The samples were hydrolyzed in 67% v/v H_2_SO_4_ for 1 h at room temperature, and then in 2 M H_2_SO_4_ at 121 °C for 1 h (h). The alditol acetate derivatives were prepared from AIRs and then determined by GC-MS (Zhou et al. 2009). The crystalline cellulose was measured with a modified Updegraff method, as previously described (Updegraff 1969).

For the saccharification assay, 1 mg of AIR was boiled to deactivate endogenous enzymes. After cooling to room temperature, the digestion was performed according to (Gao et al. 2017). The released sugars in the supernatant were measured by reading the A_540_ on an ELISA reader (Tecan) as described previously (Gao et al. 2017).

### Map-based cloning

The *sdbc1* locus was mapped and cloned using 5014 homozygous mutant F_2_ plants, which were selected from the population of *sdbc1* and 93–11. Molecular markers distributed throughout the whole rice genome were used for *sdbc1* locus rough mapping. Molecular markers for fine mapping were developed to narrow down the mutated locus to a 45 kb region on chromosome 9. The corresponding DNA fragments in this mapping region were amplified from mutants and WT plants using KOD DNA polymerase (TOYOBO, http://www.toyobo.co.jp/e/bio) and sequenced by BioSune company (http://www.biosune.com). For complementation of *sdbc1* mutant, the full-length *sdbc1* mutant coding sequence, a 2.5-kb fragment of upstream sequence from ATG and 0.2-kb fragment of downstream sequence from TGA were inserted into binary vector pCAMBIA2300 between the EcoRI and BamHI sites to generate the construct *pSDBC1::sdbc1* (*pSdbc1F*), which was introduced into WT by the Agrobacterium-mediated transformation procedure as described previously (Ye et al. 2018). A cleaved amplified polymorphism sequences (CAPS) marker for identification of *sdbc1* mutation background was also developed by using enzyme AhdI (NEB, www.neb.com).

### Expression Analysis

Total RNA was extracted from various rice tissues using TRIzol reagent (Invitrogen), as described previously (Wadsworth et al. 1988). The complementary DNA (cDNA) was synthesized from total RNA using a reverse transcriptional kit (TransGen, http://www.transgen.com.cn/). Quantitative RT-PCR was performed using relevant primers and qRT-PCR kit (TransGen, http://www.transgen.com.cn/) on a quantitative 7500 PCR system (ABI). All assays were repeated at least three times, the *OsActin1* (LOC_Os03g50885) gene was used as an internal control. The data were analyzed according to (Wong and Medrano 2005). All the primers used in this study are listed in Supplementary data at *RICE* online.

### Subcellular Localization Analysis

To observe OsCESA9 and OsCESA9^D387N^ subcellular localization, GFP was fused to their C-terminus and inserted into the pCAMBIA1300 driving by CaMV (Cauliflower mosaic virus) 35S promoter. The *N. benthamiana* leaves expressing GFP positive OsCESA9 and OsCESA9^D387N^ were observed using confocal laser scanning microscope (Leica TCS SP5).

### Yeast Two-Hybrid Assays

Yeast two-hybrid assays were performed by mating-based split ubiquitin system (mbSUS) according to (Obrdlik et al. 2004). The OsCESA9 and OsCESA9^D387N^ were cloned into *pX-NubWTgate* (182) vector, and transformed into yeast strain AP5. Transformants were selected on synthetic dropout (SD) media lacking tryptophan (T). OsCESA4 and OsCESA7 were cloned into *pMetYCgate* (184) vector, and transformed into yeast strain AP4. Transformants were selected on SD lacking leucine (L). The AP4 and AP5 suspensions were mixed and plated on YPD. After 12 h at 30 °C, cells were selected for diploids by replica plating on SD lacking tryptophan (T) and leucine (L), and incubated at 30 °C for 2–3 days. For growth assays, diploid cells were spotted on an SD medium lacking tryptophan (T), leucine (L), histidine (H) and adenine (A). Growth was monitored for 3–5 days. The β-galactosidase activity assays were performed for detecting the interaction intensity according to the manufacturer’s protocol (Takara Bio Inc.).

### SFLC Assays

Full length cDNAs of OsCESA4, OsCESA7 and OsCESA9 were amplified and the amplicons were inserted into *pCAMBIA1300-35S-Cluc-RBS* or *pCAMBIA1300-35S-HA-Nluc-RBS* vectors. The full-length cDNA of OsCESA9^D387N^ was amplified from *sdbc1* plants and cloned into *pBIB-35S-*OsCESA9^D387N^*-Flag* vector. All vectors were introduced into *Agrobacterium tumefaciens* (*A. tumefaciens*) strain GV3101. Agrobacterial strains carrying the indicated constructs were infiltrated into *N. benthamiana* leaves. The injected leaves were detached 2 days later and sprayed with 1 mM luciferin (Promega, E1605). LUC signal was captured using a cooled CCD-imaging apparatus (Berthold, LB985). LUC activity was measured as previously described (Chen et al. 2008).

### BiFC Assays

For the BiFC assays, the full length cDNAs of OsCESA4,OsCESA7, OsCESA9 and OsCESA9^D387N^ were cloned into serial pSPY vectors containing either N- or C-terminal enhanced yellow fluorescent protein fragments via Gateway cloning technology. These constructs were then introduced into *A. tumefaciens* strain GV3101 and co-infiltrated into the *N. benthamiana* leaves. The injected leaves were detached after 2 days to observe fluorescence with a confocal laser-scanning microscope (TCS SP5; Leica).

### Western Blot

The extraction of total membrane protein and separation of the plasma membrane (PM) and endomembrane fractions were performed as previously described (Zhang et al. 2009). Two grams of 4-week-old WT and *sdbc1* plants were ground to fine power in liquid nitrogen and extracted with ice-cold extraction buffer (25 mM Tris-HCl, pH = 7.5, 0.25 M sucrose, 2 mM EDTA, 2 mM DTT, 15 mM β-mercaptoethanol, 10% Glycerol, and proteinase inhibitor cocktail). After centrifugation at 10,000 g for 20 min at 4 °C, the supernatant was ultracentrifuged at 100,000 g for 1 h at 4 °C to obtain a microsomal pellet. The microsomal proteins were further separated by suspension in the fractionation buffer (5 mM Na_2_HPO_4_-NaH_2_P_4_, pH 7.2, 0.25 M sucrose, 1 mM DTT, 6.2% PEG3350 and 6.2% Dextran T500) and centrifugation at 8000 g for 10 min at 4 °C. The proteins in the PEG and DEX fractions were separately collected and concentrated at 100,000 g for 1 h. The pellets were dissolved in suspension buffer (2 mM Tris, pH 6.5, 1 mM DTT and 0.25 M sucrose). Ten micrograms of protein was run on an SDS–PAGE gel and probed with corresponding polyclonal antibodies and the secondary antibody HRP-conjugated anti-rabbit IgG (Sigma). The reactions were detected by the ECL Plus Western Blotting Detection System kit (GE Healthcare). The antibodies of Anti-PIP1s and Anti-BiP2 were purchased from Agrisera (http://www.agrisera.com). Generations of CESA4-, CESA7- and CESA9- specific antibodies were described previously (Zhang et al. 2009).

### Salt Stress

The WT, *sdbc1* and F1 plants were grown under normal hydroponic culture (1.44 mM NH_4_NO_3_, 0.3 mM NaH_2_PO_4_, 0.5 mM K_2_SO_4_, 1.0 mM CaCl_2_, 1.6 mM MgSO_4_, 0.17 mM Na_2_SiO_3_, 50 μM Fe-EDTA, 0.06 μM (NH_4_)_6_Mo_7_O_24_,15 μM H_3_BO_3_, 8 μM MnCl_2_, 0.12 μM CuSO_4_, 0.12 μM ZnSO_4_, 29 μM FeCl_3_, and 40.5 μM citric acid [pH 5.5]) after 1 week following germination. The seedlings were kept under these conditions for 1 month following which half of the plants were treated with 200 mM NaCl, and the remaining plants were maintained as before. The samples for RNA extraction from control and salt treated plants were collected at 48 h after salt stress had been applied to half of the plants. Root and shoot samples were harvested for biomass and ion content measurements following 1 week after the salt stress treatment.

### DAB Staining for H_2_O_2_

3,3′-Diaminobenzidine (DAB) staining was performed as previously described (Fang et al. 2018). Plant leaves were detached and immersed in 1% solution of DAB in deionized water (pH = 3.8). After vacuum infiltration for 30 min, the samples were incubated at room temperature for 24 h in the dark. After which, leaves were bleached by immersing in boiling ethanol to remove chlorophyll and reveal the brown spots, which are indicative of the DAB reaction with H_2_O_2_.

### Determination of the MDA Content and Relative Electrical Conductivity

The content of MDA was measured as previously described (Ouyang et al. 2010). In brief, about 1 g leaves was homogenized in 10 mL of 10% trichloroacetic (TCA) followed by centrifugation at 5000 g for 10 min. Subsequently, 2 mL of the supernatant was mixed with an equal volume of thiobarbituric acid (TBA) and boiled for 15 min and the quickly cooled on ice. The absorbance valus at wavelength of 450, 532 and 600 nm were measured on a plate reader (Tecan). The final quantity of MDA (μmol/L) was calculated by using the following formula: 6.45 * (A_532_ – A_600_) – 0.56 * A_450_.

The relative electrical conductivity (REC) was measured according to (Cao et al. 2007). The washed leaves were cut into 0.5 cm pieces and immersed in a 50 mL test tube containing 25 mL deionized water for 30 min, and then the conductivity of the solution was measured using a conductivity meter (DDS-11A). After boiling the samples for 10 min, their conductivity was measured again when the solution was cooled to room temperature. The relative electrical conductivity (REC) was calculated by using the following formula: REC = C_1_/C_2_ *100%. Where C_1_ and C_2_ are the electrolyte conductivities measured before and after boiling, respectively.

### Measurements of Ion Contents

Measurements of ion concentration in rice tissues were performed as described elsewhere (Fang et al. 2018). The samples of various tissues were treated at 105 °C for 1 h. Powered samples were digested with ultrapure nitric acid for 1 day, and the digested samples were boiled at 95 °C for 10 min three times. Ion contents were determined using an inductively coupled plasma optical emission spectrometer (ICP-MS, Agilent 7700 series, USA).

### Statistical Analysis

All data were analyzed in SPSS version 20.0. Student’s *t*-test was used to compare pairs of means, whilst comparisons between multiple groups were performed using ANOVA followed by Duncan’s multiple range test.

### Accession Numbers

Sequence data used in this manuscript can be found in the rice genome annotation database (http://rice.plantbiology.msu.edu) under the following accession numbers: *OsCESA4* (LOC_Os01g54620), *OsCESA7* (LOC_Os10g32980), *OsCESA9* (LOC_Os09g25490), *OsActin1* (LOC_Os03g50885), *OsSOS1* (LOC_Os12g44360), *OsKAT1* (LOC_Os01g55200), *OsHKT1;5* (LOC_Os01g20160), *OsHAK10* (LOC_Os06g42030), *OsNHX1* (LOC_Os07g47100), *OsHKT1;1* (LOC_Os04g51820), *OsHKT2;1* (LOC_Os06g48810).

## Results

### *Sdbc1* Is a Semi-Dominant Brittle Culm Mutant and Shows a Reduction in Mechanical Strength and Cellulose Content

**Fig. 1:**
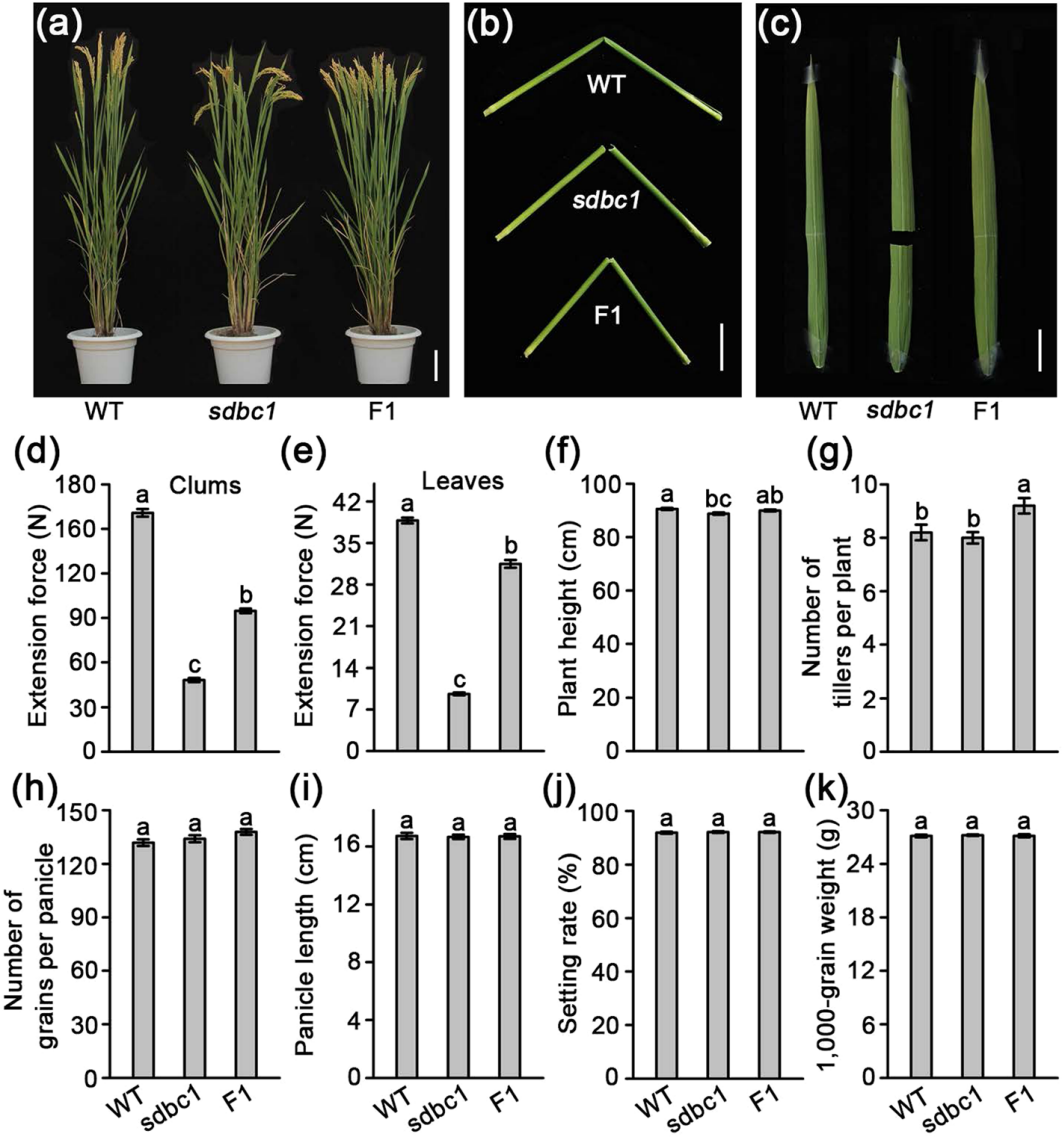
*sdbc1* mutant identification and agronomic trait observation. **a** Four month old wild type (WT), *sdbc1* homozygous (*sdbc1*) and *Sdbc1* heterozygous (F1) plants. Bar = 10 cm. **b**, **c** Brittleness of culms (Bar = 5 cm) and leaves (Bar = 3 cm). **d**, **e** Measurements of the extension force of internodes and leaves. **f** Plant height. **g** Tiller number **h** Number of grains per panicle. **i** Panicle length. **j** Seed setting rate. **k** 1000-grain weight. Error bars represent SE (*n* = 50). Different letters denote significant differences (*P* < 0.05, Duncan’s multiple range test)

A novel *bc* mutant was isolated from the *japonica* cultivar, Wuyunjing7 by treatment with a heavy ion beam. Unlike most other *bc* mutants previously reported (Li et al. 2018; Li et al. 2017; Song et al. 2013; Tanaka et al. 2003; Wang et al. 2016a; Wang et al. 2012), the crossing of this novel brittle culm mutant with normal culm phenotype cultivars, resulted in all of the F_1_ plants exhibiting the mild brittle culm phenotypes. The F_2_ progeny from self-fertilized F_1_ plants showed an approximately 1: 2: 1 segregation of normal: mild brittle: easily brittle culm phenotypes, which indicated that the mutant is a semi-dominant brittle culm (*sdbc*) mutant, hence we called it *sdbc1* (Table S1). The homozygous *sdbc1* mutant (termed as *sdbc1*) plants showed easily broken culms and leaves, but the WT/*sdbc1* heterozygous F_1_ (termed as F1) plants only exhibited mild brittle culm phenotypes. Except for the brittle culm phenotype, both were morphologically indistinguishable from the WT during the mature stage (Fig. 1a, b, c). In the seedling stage, *sdbc1* plants showed an easily broken and slender phenotype, but the F1 plants only showed a slender but not easily broken phenotype (Figure S1). We quantitatively measured the force required to break the second internode of the culms and the flag leaves in the WT, *sdbc1*, and F1 plants. The force to break the culms of *sdbc1* and F1 plants was reduced to about 30% and 60% of that to break the culms of the WT, respectively (Fig. 1d), and the force to break the leaves of *sdbc1* was only about a quarter of that required to break WT leaves, but the force to break the leaves of the F1 plants was only reduced by about 19% (Fig. 1e). Although mechanical strength was affected in *sdbc1* and F1 plants, others traits, such as plant height (Fig. 1f), tillers per plant (Fig. 1g), grains per panicle (Fig. 1h), panicle length (Fig. 1i), seed setting rate (Fig. 1j) and 1000-grain weight (Fig. 1k), showed no significant difference compared with the WT.

**Fig. 2:**
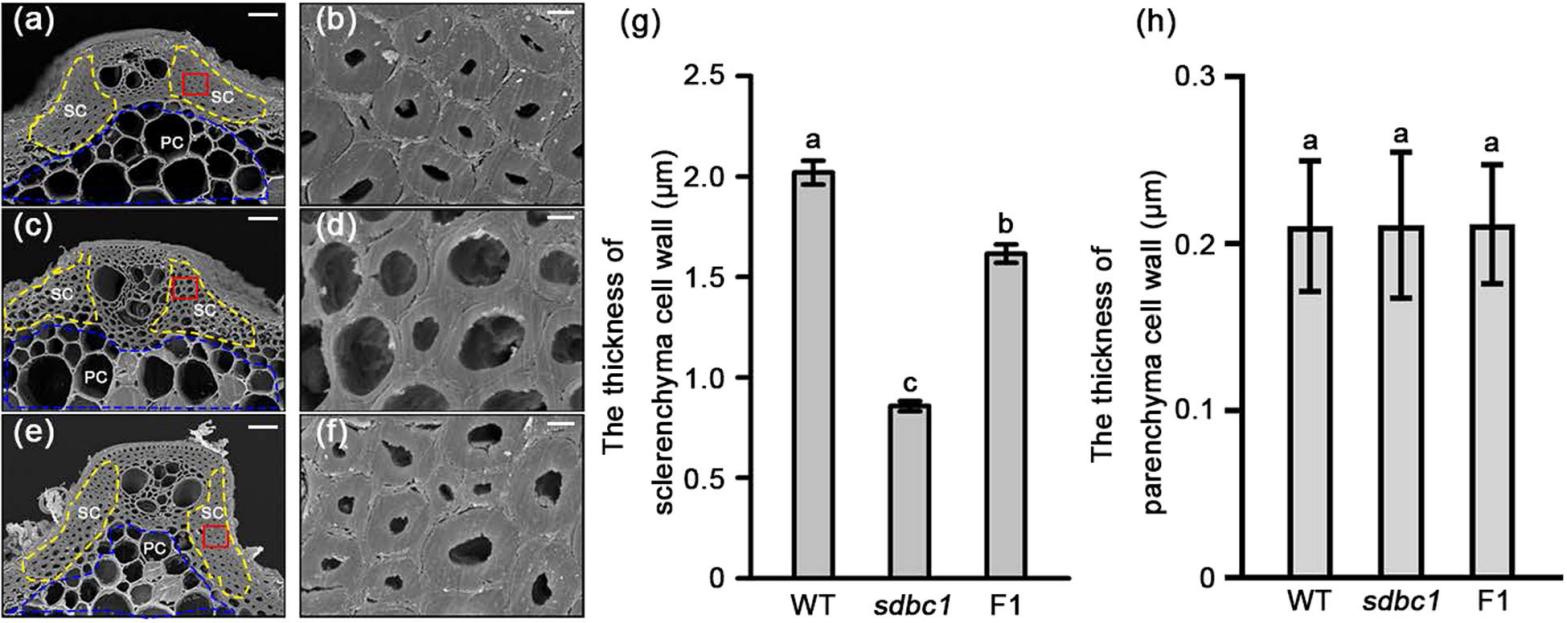
Scanning electron micrographs of the sclerenchyma cell walls of WT (**a**), *sdbc1* (**c**) and F1 plant (**e**). **b**, **d** and **f** are enlargements of the red boxed areas in **a**, **c** and **e**, respectively. Bars = 20 μm (**a**, **c** and **e**) and 2 μm in (**b**, **d** and **f**). **g** The thickness of sclerenchyma cell walls. **h** The thickness of parenchyma cell walls. SC: sclerenchyma cell, indicated by the yellow dash line; PC: parenchyma cell, indicated by the blue dash line. Error bars represent SE (*n* = 30). Different letters denote significant differences (P < 0.05, Duncan’s multiple range test)

To understand the underlying cause of the observed brittleness, scanning electron microscopy (SEM) was performed to observe the second internode cross-sections of the WT, *sdbc1* and F1 plants. The observations revealed that the sclerenchyma cell walls, the thickness of which is highly correlated with mechanical strength, were obviously thickened in the WT plants (Fig. 2a, b, g). In contrast, the thickness of sclerenchyma cell walls in the *sdbc1* plants was reduced obviously (Fig. 2c, d, g). The thickness of the sclerenchyma cell walls in the F1 plants was between the WT and *sdbc1* (Fig. 2e, f, g). No obvious differences were observed in the parenchyma cell walls (Fig. 2a, c, e, h). The defects in mechanical strength and wall structure suggested that the cell wall composition in the *sdbc1* and F1 plants may be altered. We therefore examined the cell wall composition in the second internodes of the WT, *sdbc1* and F1 plants at the mature stage. As shown in Table 1, the cellulose content of the *sdbc1* and F1 internodes was decreased by about 30% and 14% that of the WT, respectively, whereas the neutral sugar content derived from non-cellulosic polysaccharides was generally increased in *sdbc1* and F1 samples, especially for arabinose (Ara) and xylose (Xyl), the two major sugars of hemi-cellulose arabinoxylan at a significantly high level (Table 1), we also detected the total hemi-cellulose content of the *sdbc1* and F1 internodes higher than that of WT (Figure S2a), but pectin and starch content were not significant different (Figure S2b, c).

**Table 1:** Cell wall composition analysis of internodes of wild type, *sdbc1* and F1 plants

Sample	Rha	Fuc	Ara	Xyl	Man	Gal	Glu	Cellulose
Wild type	1.73 ± 0.01	0.83 ± 0.01	32.39 ± 0.42	191.53 ± 6.18	1.41 ± 0.01	12.17 ± 0.21	58.63 ± 0.33	463.26 ± 3.89
*sdbc1*	2.26 ± 0.03*	0.84 ± 0.02	46.18 ± 1.86*	295.33 ± 7.43*	1.34 ± 0.01	15.32 ± 0.98*	71.26 ± 1.95*	323.16 ± 4.62*
F1	1.96 ± 0.01	0.85 ± 0.01	38.43 ± 0.36	265.22 ± 5.27*	1.39 ± 0.01	13.69 ± 0.47*	64.54 ± 1.36*	398.93 ± 4.45*

The results are means ±SE of five independent assays. Each wall component was calculated as mg·g^− 1^ of alcohol-insoluble cell-wall residue

*Significant difference (*t*-test at *P* < 0.01) with respect to wild-type

Taken together, the *Sdbc1* is a semi-dominant brittle culm mutant, and its defects in mechanical strength are correlated with thin sclerenchyma cell walls and lower cellulose content and higher hemi-cellulose content in the *Sdbc1* mutant.

### A Conserved-Site Mutation in OsCESA9 Results in *Sdbc1* Phenotypes

**Fig. 3:**
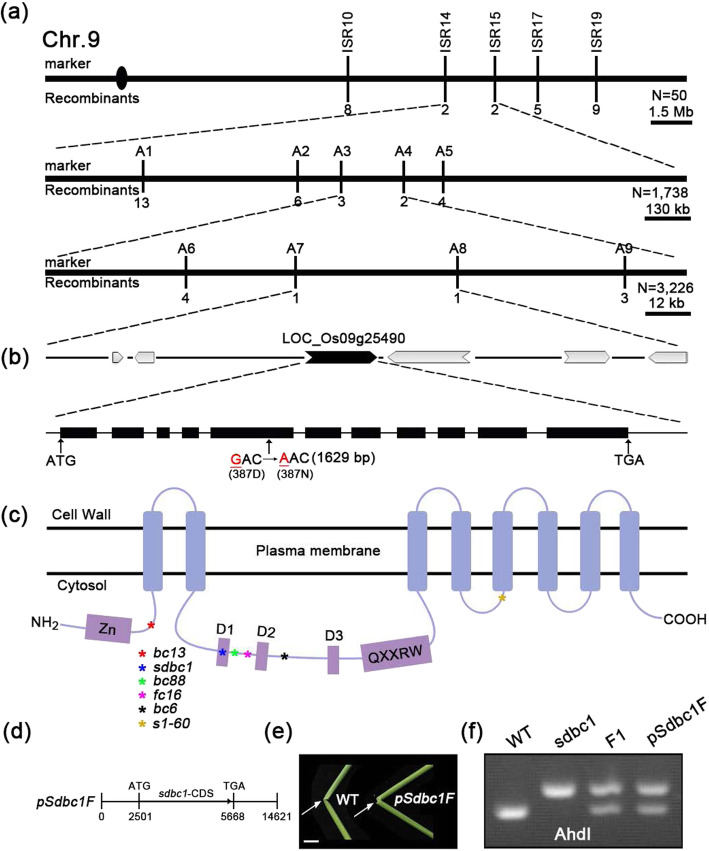
Map-based cloning of the *SDBC1* gene. **a** The sdbc1 locus was mapped to the region between markers ISR14 and ISR15 on chromosome 9 and further narrowed to an approximately 45 kb region between markers A7 and A8. Vertical lines represent the positions of molecular markers and the number of recombinants. **b** 6 predicted ORFs within the fine mapping region and sequencing analysis revealed a point mutation that results one amino acid change at the 387th residue. **c** Protein structure of OsCESA9. Different color asterisks represent different *OsCESA9* alleles. **d** A construct for complementary assay. **e** Folding the internodes of rice plants (indicated by the arrows) to show the reduced mechanical property in the complemented plants, Bar = 2 cm. **f** A CAPS marker (digested by AhdI) was developed to distinguish the WT and *sdbc1* background

To investigate the molecular basis of the above phenotypes, a map-based cloning approach was performed to isolate the *SDBC1* gene. We used the F_2_ population generated by crossing *sdbc1* with 93–11, a WT polymorphic *indica* variety. The *sdbc1* locus was located between molecular markers ISR14 and ISR15 on chromosome 9, and further pinpointed within an approximate 45-kb region between markers A7 and A8 (Fig. 3a). The 45-kb region contains 6 putative open-reading frames (ORFs), in which the most possible candidate ORF *Os09g25490* that encodes OsCESA9 involved in secondary cell wall cellulose biosynthesis was selected for sequencing (Fig. 3b). Sequencing of this ORF in *sdbc1* revealed one base pair mutation at position 1629, which changes GAC to AAC and causes a substitution at the 387th amino acid residue from aspartic acid (Asp, D) to asparagine (Asn, N) (Fig. 3b). The sequencing atlas showed that mild brittle culm plants were bimodal at the mutation site (Figure S3), which further suggested that *Sdbc1* was a semi-dominant brittle culm mutant. This mutation is at the first conserved aspartic acid residue domain (Fig. 3c), which is fully conserved in all of the CESA family proteins (Figure S4). To confirm that *Os09g25490* corresponds to the *sdbc1* locus, a 5.6-kb DNA fragment containing the 2.5-kb putative promoter and the coding region from *sdbc1* was cloned into vector *pCAMBIA2300* to generate the plasmid *pSdbc1F* (Fig. 3d), which was introduced into the WT plants. All 10 of the transgenic lines showed the mild brittle culm phenotype (Fig. 3e). The mutated site also was confirmed by a CAPS marker, digested by AhdI (Fig. 3f). We therefore concluded that *Os09g25490* is *SDBC1*.

### The CESA9^D387N^ Mutation Enhances Biomass Saccharification

**Fig. 4:**
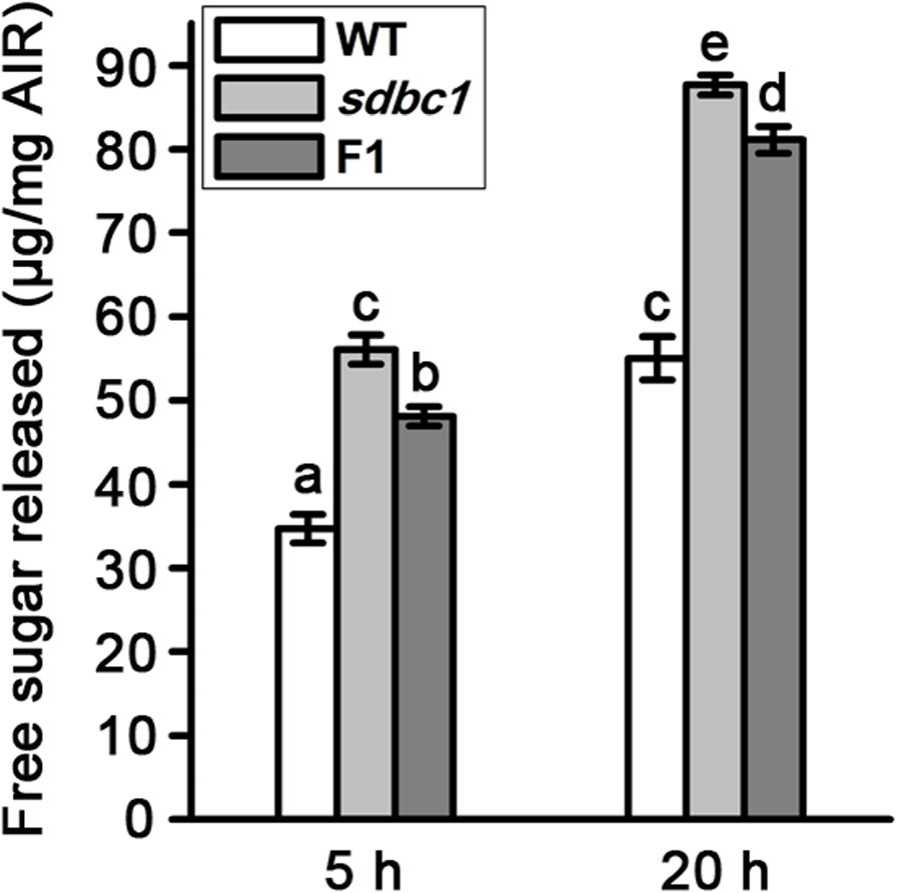
Saccharification analysis of the wall residues from WT, *sdbc1* and F1 internodes. The wall residues were treated with enzyme mixture for 5 and 20 h. Error bars indicate SE from the mean of three replicates. Different letters denote significant differences (P < 0.05, Duncan’s multiple range test)

In recent years, rice straw has been highlighted as an important material for biofuel production, the high cellulose content and crystallinity determine lignocellulose recalcitrance, leading to costly biomass processing (Alam et al. 2019; Himmel et al. 2007). As previously reported (Li et al. 2017), the *Osfc16* mutant with substitutions (W481C, P482S) at the P-CR conserved site in OsCESA9 can improve enzymatic saccharification. We detected whether the CESA9^D387N^ mutation also enhances biomass saccharification. We examined the saccharification efficiency of lignocellulosic material derived from WT, *sdbc1*, and F1 plants. The sugar yields were significantly higher in *sdbc1* and F1 than that of WT (Fig. 4). These results suggest that the OsCESA9^D387N^ mutation can enhance biomass enzymatic saccharification.

### The CESA9^D387N^ Mutation Does Not Affect the Expression Pattern of *OsCESA9* or its Subcellular Localization

**Fig. 5:**
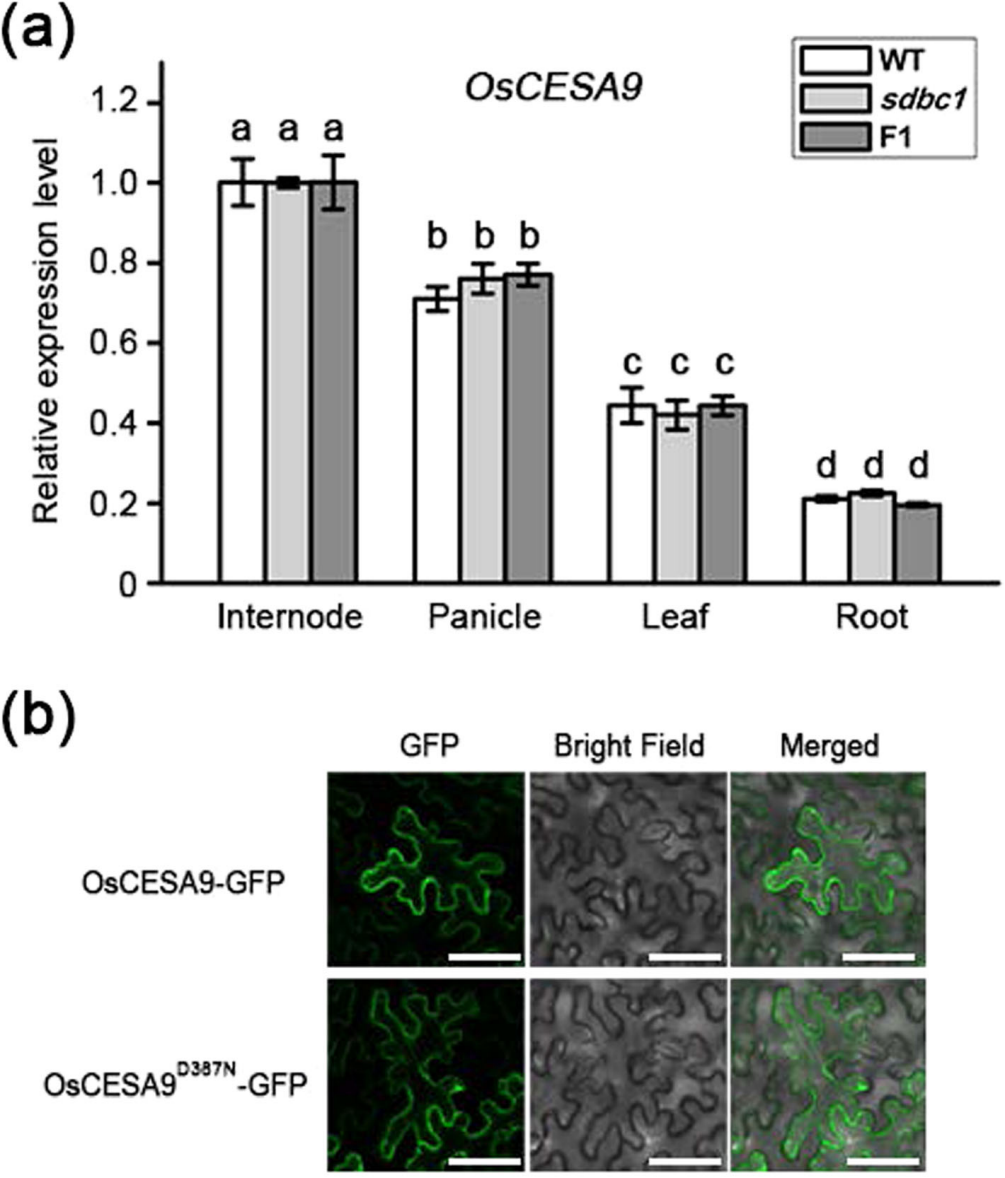
OsCESA9^D387N^ mutation does not affect its expression pattern and subcellular localization. **a** The expression level of O*sCESA9* in various rice organs of WT, *sdbc1* and F1 plants. The *Actin1* gene was used as an internal control. **b** The subcellular localization of OsCESA9 and OsCESA9^D387N^. Full-length OsCESA9 and OsCESA9^D387N^ fused with green fluorescent protein (GFP) were expressed in *N. benthamiana* leaves, Bars = 60 μm

The missense mutation (D387N) in *sdbc1* occurs at the first conserved aspartic acid residue. Among the various mutations in OsCESA isoforms, the mutation of this aspartic acid has not been reported. To determine the effect of this important amino acid residue at the molecular level, we first detected whether the expression level and pattern of *OsCESA9* had been affected in *sdbc1* and F1 plants. Quantitative real-time polymerase chain reaction (qRT-PCR) analysis revealed that the expression level and pattern of *OsCESA9* had not been affected in the WT, *sdbc1* and F1 plants (Fig. 5a). Given that OsCESA9 needs to interact with OsCESA4 and OsCESA7 to form functional CSCs for secondary cell wall cellulose synthesis, the expression levels and patterns of *OsCESA4* and *OsCESA7* were also detected by qRT-PCR. There were no obvious differences in the WT, *sdbc1* and F1 plants (Figure S5).

The correct subcellular localization of a protein is important for it to function normally. To determine whether the conserved-site mutation in OsCESA9 affects its subcellular localization, we expressed the WT form OsCESA9-GFP and mutation form OsCESA9^D387N^-GFP in *Nicotiana benthamiana* leaves. Confocal laser scanning microscope observation revealed that fluorescent signals of OsCESA9-GFP and OsCESA9^D387N^-GFP were detected in the plasma membrane (Fig. 5b), which was consistent with the subcellular localization of OsCESA9.

Taken together, the CESA9^D387N^ mutation does not affect the expression pattern of *OsCESA9* or change its protein localization.

### OsCESA9^D387N^ Can Compete with OsCESA9 to Interact with OsCESA4 and OsCESA7

**Fig. 6:**
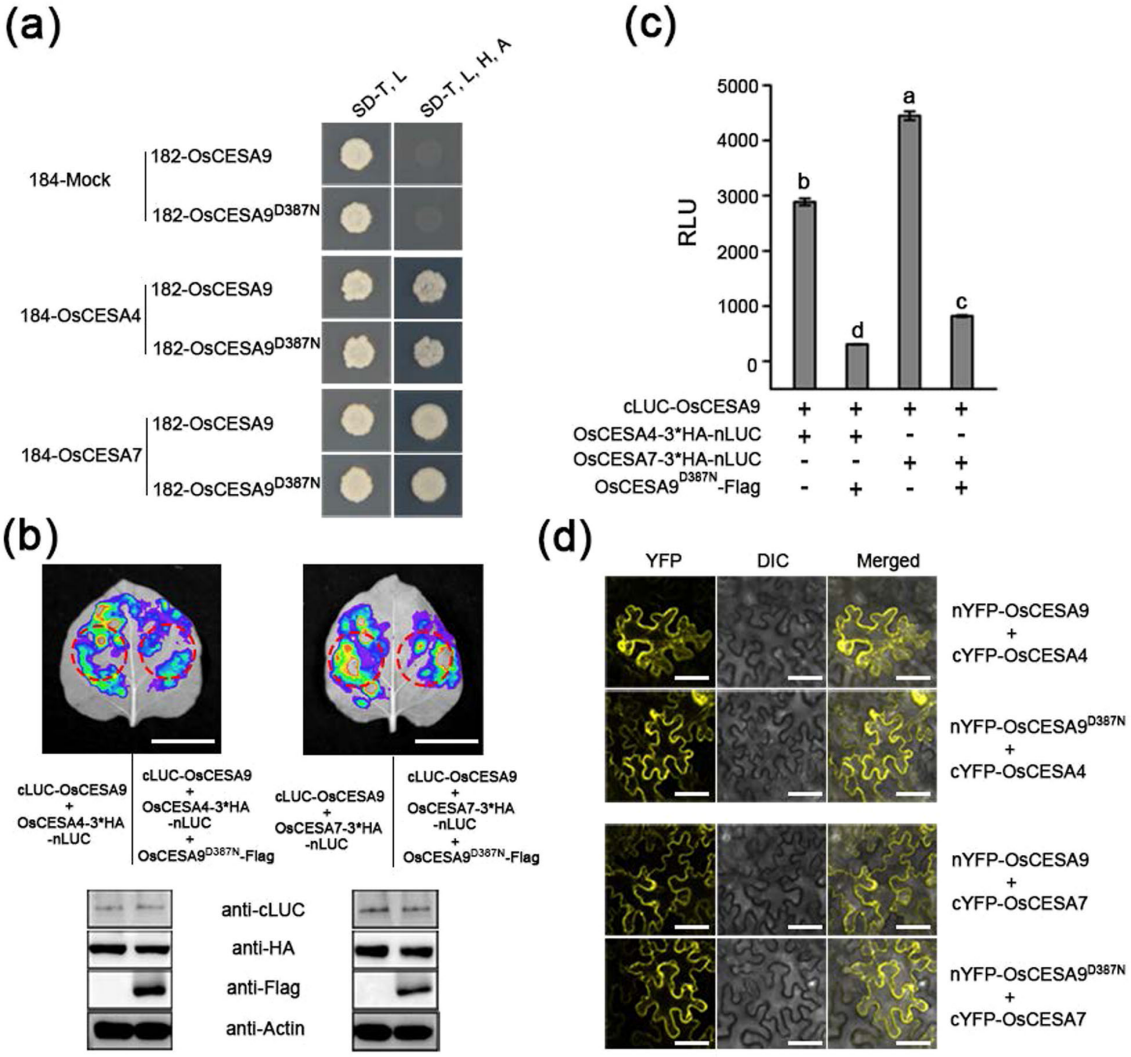
OsCESA9^D387N^ compete with OsCESA9 for interaction with OsCESA4 and OsCESA7. **a** Both OsCESA9 and OsCESA9^D387N^ can interact with OsCESA4 and OsCESA7 in yeast. 182-OsCESA9 and 182-OsCESA9^D387N^ are the bait plasmid containing full length cDNA of OsCESA9 and OsCESA9^D387N^, respectively. 184-OsCESA4 and 184-OsCESA7 are the prey plasmid containing full length cDNA of OsCESA4 and OsCESA7, respectively. 184-Mock, empty prey plasmid. SD/−T -L, SD medium lacking tryptophan and leucine; SD/−T -L -H -A, SD medium lacking tryptophan, leucine, histidine, and adenine. **b**, **c** Split firefly luciferase complementation (SPLC) assays showing the interactions of OsCESA9 with OsCESA4 and OsCESA7 were weakened in the presence of OsCESA9^D387N^ in *N. benthamiana* leaves, Bars = 2 cm. The western blotting results in (**b**) indicating all constructs were agroinfiltrated into *N. benthamiana* leaves have been expressed. **c** The relative luminescence unit (RLU) values were measured by using the red circle (**b**) for crude enzyme extraction. Different letters denote significant differences (P < 0.05, Duncan’s multiple range test). **d** BiFC assays indicate that OsCESA9^D387N^ direct interact with OsCESA4 and OsCESA7 on the plasma membrane, consist with its WT form OsCESA9, Bars = 60 μm

As mentioned above, OsCESA9 needs to interact with OsCESA4 and OsCESA7 to form functional CSCs. To determine whether OsCESA9^D387N^ affects the interaction with OsCESA4 and OsCESA7, the mating-based split ubiquitin system (mbSUS) for detecting membrane protein interaction was performed. The yeast two-hybrid (Y2H) assay showed that OsCESA9 and OsCESA9^D387N^ could directly interact with OsCESA4 and OsCESA7 in vitro (Fig. 6a), and the β-galactosidase activity assay indicated that there was no significant change in the interaction intensity of OsCESA9^D387N^ with OsCESA4 and OsCESA7, compared with OsCESA9 (Figure S6). Thus, OsCESA9^D387N^ can also interact with OsCESA4 and OsCESA7 to form CSCs.

Given that the *OsCESA9*^*D387N*^ is a semi-dominant mutation and OsCESA9^D387N^ can interact with OsCESA4 and OsCESA7, we speculated that the mutation OsCESA9^D387N^ will compete with WT OsCESA9 for physical interaction with OsCESA4 and OsCESA9. To verify this hypothesis, the split firefly luciferase complementation (SFLC) assays were performed, the results show that the interaction of OsCESA4 or OsCESA7 with OsCESA9 is stronger in the absence of OsCESA9^D387N^ than that of in the presence of OsCESA9^D387N^, even though they all had the same level of protein abundance (Fig. 6b). We further quantified interaction intensity and showed that the LUC activity significantly decrease in the presence of OsCESA9^D387N^ (Fig. 6c). These suggest that the interactions of OsCESA9 with OsCESA4 and OsCESA7 were weakened in the presence of OsCESA9^D387N^ (Fig. 6b, c). Thus, we reasoned that OsCESA9^D387N^ is able to compete with OsCESA9 for interaction with OsCESA4 and OsCESA7. The CSCs are located in the plasma membrane where they perform their function. We further tested whether OsCESA9^D387N^ altered the subcellular localization of the protein complex with OsCESA4 and OsCESA7. Bimolecular fluorescence complementation (BiFC) assays were performed to verify that OsCESA9^D387N^ directly interacts with OsCESA4 and OsCESA7 on the plasma membrane, consistent with its WT form OsCESA9 (Fig. 6d).

In combination, these results suggest that OsCESA9^D387N^ can compete with OsCESA9 for interaction with OsCESA4 and OsCESA7, but it does not affect the subcellular localization of the OsCESA4/7/9^D387N^ complex.

### The OsCESA9^D387N^ Mutation Does Not Affect Secondary Wall CSC Trafficking

**Fig. 7:**
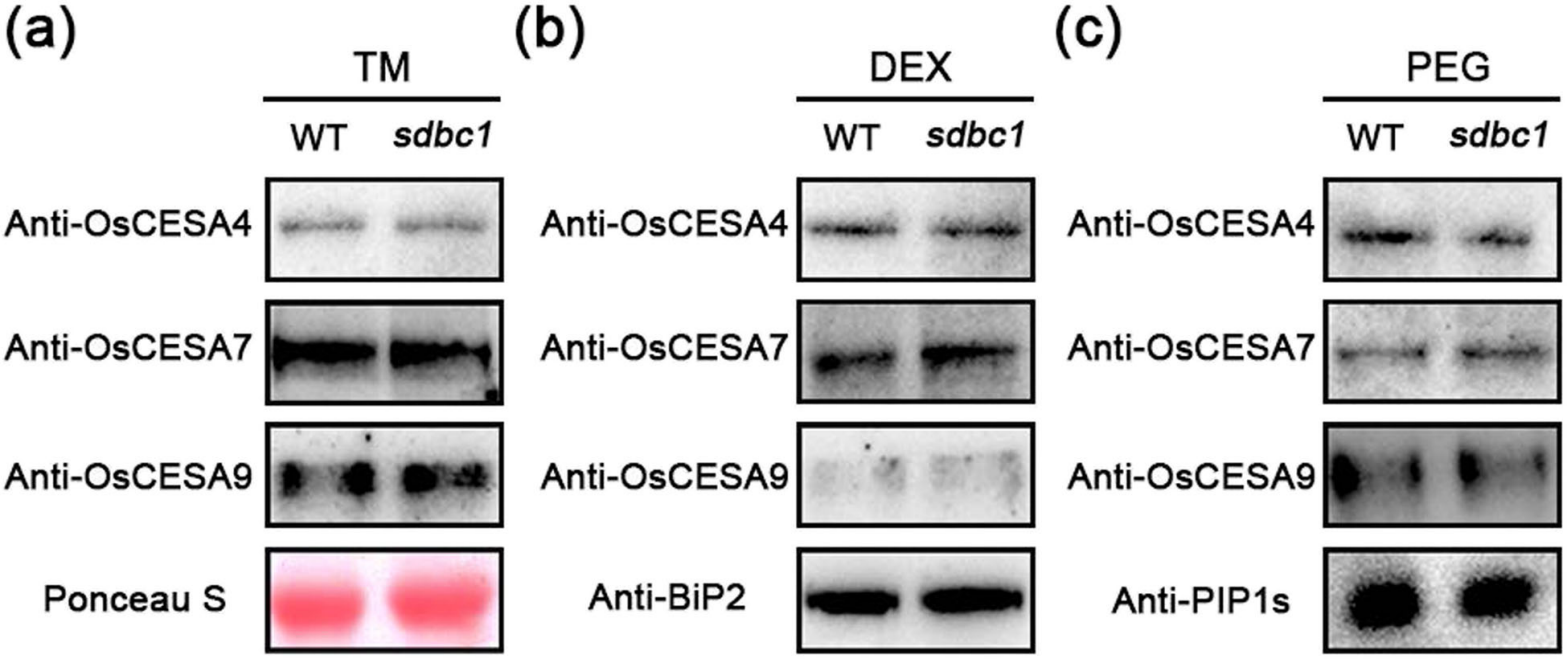
The OsCESA9^D387N^ mutation does not affect secondary cell wall CSC trafficking. **a** Western blotting analysis of OsCESA4, OsCESA7 and OsCESA9 with their polyclonal antibodies in microsomal pellet between WT and *sdbc1* plants. Ponceau S, loading control; TM, total membrane. **b** and **c** Western blotting analysis of OsCESA4, OsCESA7 and OsCESA9 in DEX (endomembrane) and PEG (plasma membrane) fractioned membrane proteins extracted from WT and *sdbc1* plants. DEX, the endomembrane fraction; PEG, the plasma membrane fraction; Anti-BiP2 and Anti-PIP1s antibodies are used to label the marker protein in the endomembrane and plasma membrane, respectively. Western blotting analysis has been repeated for at least three times

Our current understanding of cellulose synthesis suggests that CESAs are assembled into CSCs in either the endoplasmic reticulum (ER) or the Golgi apparatus and trafficked by vesicles to the plasma membrane (PM) (McFarlane et al. 2014). We aimed to determine whether the missense mutation in *sdbc1* affects the intracellular trafficking of OsCESA4, OsCESA7 and OsCESA9. First, the amount of OsCESA4, OsCESA7 and OsCESA9 proteins detected by western blot analysis did not differ much (Fig. 7a). We then examined the distribution and/or abundance of OsCESA4, OsCESA7 and OsCESA9 between the PM and endo-membrane systems in WT and *sdbc1* plants. We therefore separated proteins of the plasma membrane (PEG fraction) and endo-membranes (DEX fraction) and probed them with corresponding polyclonal antibodies. The western blot results showed that there was no significant difference in the protein levels of OsCESA4, OsCESA7 and OsCESA9 between the WT and *sdbc1* plants in the endomembrane (Fig. 7b) or PM (Fig. 7c). Thus, the OsCESA9^D387N^ mutation does not affect secondary cell wall CSC protein intracellular trafficking or abundance at the PM.

### OsCESA9/OsCESA9^D387N^ Heterozygous Plants Have Better Salt Tolerance

**Fig. 8:**
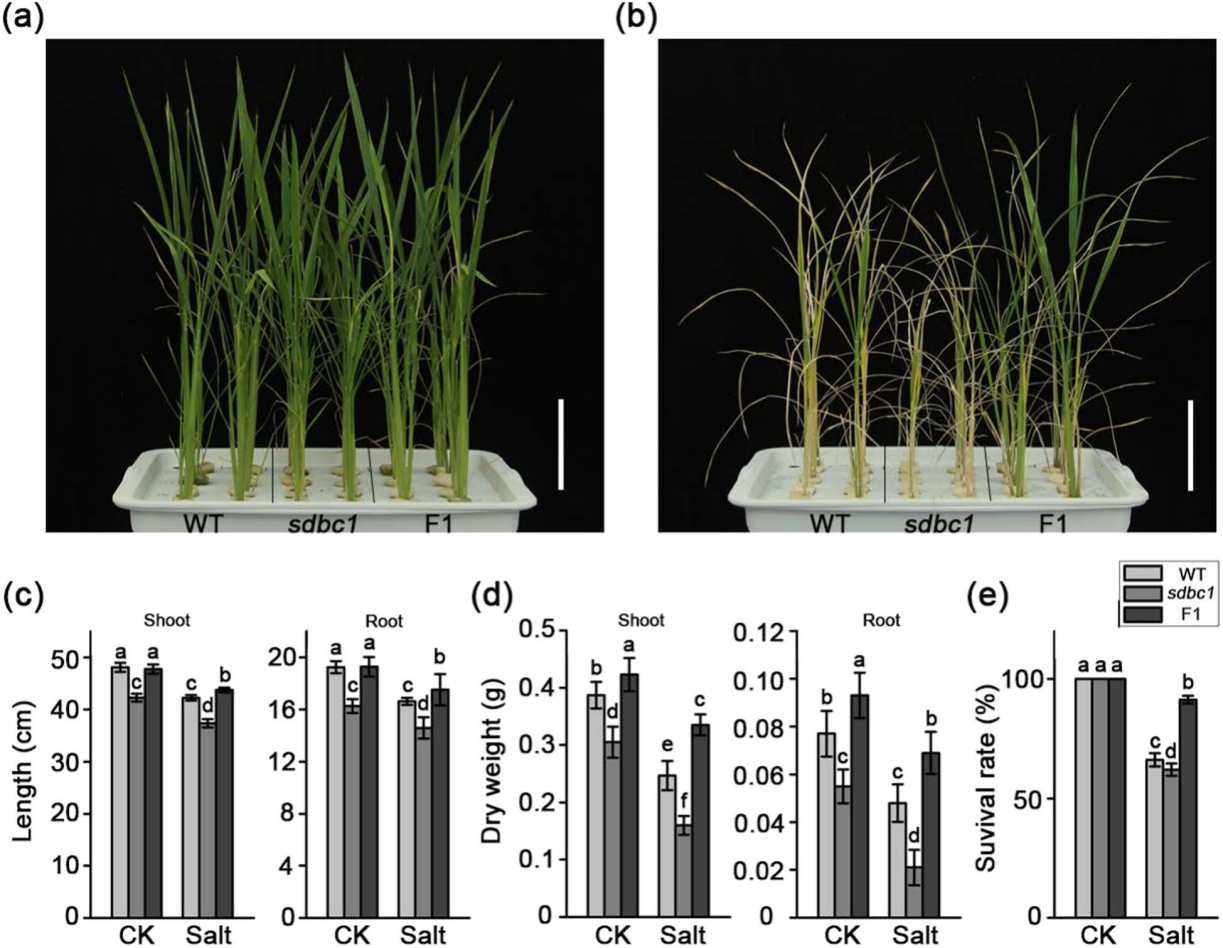
The *Sdbc1* heterozygous (F1) plants have more tolerance to salt. **a** and **b** are the phenotypes of WT, *sdbc1* and F1 plants before and after slat stress, respectively. **c** The shoot (left) and root (right) lengths for WT, sdbc1 and F1 plants under control and salt stress conditions. **d** The shoot (left) and root (right) biomasses per plant for WT, *sdbc1* and F1 plants under control and salt stress conditions. **e** The survival rates of the WT, *sdbc1* and F1 plants after salt stress treatment. Error bars indicate the SE of three biological repeats. Different letters denote significant differences (P < 0.05, Duncan’s multiple range test)

Soil salinity is one of the major stresses adversely affecting plant growth and crop productivity (Munns and Gilliham 2015). As previously reported, modulating cell wall cellulose synthesis may be one of the main adaptations of plants to osmotic stresses, such as salinity stress (Chen et al. 2005). To determine whether the OsCESA9^D387N^ mutation improves plant tolerance to salt, salinity stress was applied to *sdbc1* and F1 plants. One-month-old WT, *sabc1* and F1 plants were treated with either 0 or 200 mM NaCl for 1 week. These plants grew normally under normal hydroponic culture (Fig. 8a). However, the F1 plants grown in 200 mM NaCl demonstrated significant differences in salt sensitivity in terms of seedling growth (Fig. 8b). Moreover, the F1 plants exhibited a smaller decline in seedling height and biomass under salt stress than the WT and *sabc1* plants (Fig. 8c, d). Additionally, the survival rates of the F1 plants 1 week after 200 mM NaCl treatment were significantly higher than those of the WT and *sdbc1* plants (Fig. 8e). To further confirm phenomenon, three-week-old WT, sdbc1 and F1 plants were subjected to 100 mM NaCl for one week, the survival rates of the F1 plants were significantly higher than those of the WT and *sdbc1* plants (Figure S7). Together, these results suggested that the *OsCESA9/OsCESA9*^*D387N*^ heterozygous genotype might enhance salt stress tolerance.

Physiological analyses were performed to reveal some mechanism insights into the salt tolerance phenotype of the *OsCESA9/OsCESA9*^*D387N*^ heterozygous plants. Salt stress usually causes damage in plants by producing reactive oxygen species (ROS), such as H_2_O_2_. To detect whether ROS accumulation is altered in F1 plants after salt stress, the leaves of WT, *sdbc1* and F1 plants were staining with 3,3′-Diaminobenzidine (DAB) to visualize H_2_O_2_ content. H_2_O_2_ levels were extremely low in all plants before salt treatment (Figure S8a). However, after 200 mM NaCl treatment, the F1 plants had very few brown H_2_O_2_ spots within the total leaf area, whereas the WT and *sdbc1* exhibited more brown areas than those of the F1 plants (Figure S8a). Relative electrical conductivity (REC) is an indicator of cell membrane injury, and malondialdehyde (MDA) is an indicator of oxidative attack on membrane lipids. Leaves from F1 plants accumulated significantly lower MDA content and REC than those of WT and *sdbc1* plants under salt stress (Figure S8b, c). Proline accumulation is linked with stress tolerance, therefore we detected proline content among these plants. Although proline content increased dramatically under salt stress, there was more in F1 plants compared with that in WT and *sdbc1* plants (Figure S8d). These results suggested that the *OsCESA9/OsCESA9*^*D387N*^ heterozygous plants show more salt tolerance.

**Fig. 9:**
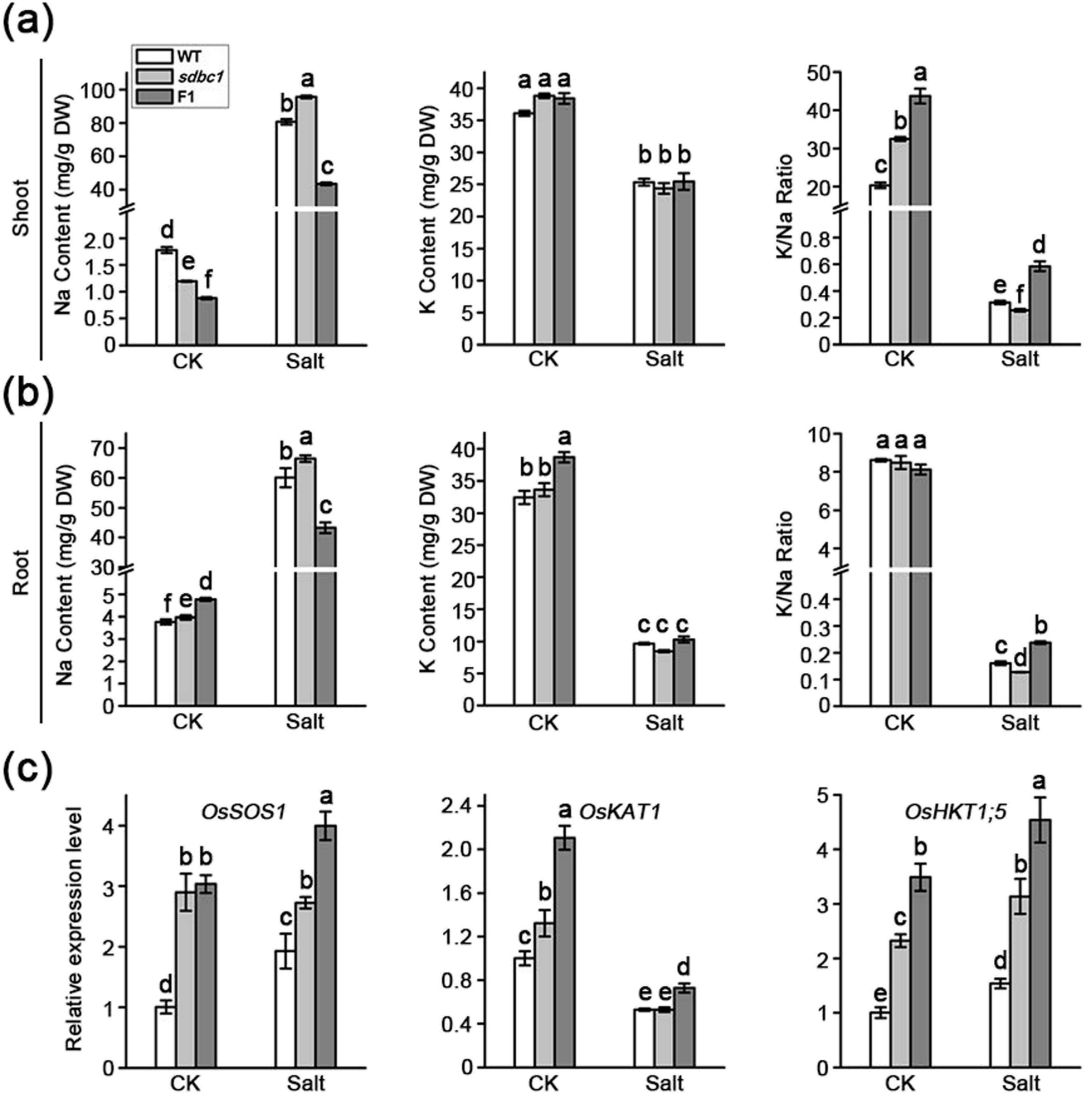
OsCESA9^D387N^ mutation effects Na^+^ and K^+^ homeostasis. **a** and **b** Na^+^ content (left), K^+^ content (middle) and K^+^/Na^+^ ratio (right) in the shoots and roots of WT, *sdbc1* and F1 plants, respectively. Different letters denote significant differences. **c** Expression levels of genes that encode for Na^+^ and K^+^ transporters in WT, *sdbc1* and F1 plants. The *Actin1* gene was used as an internal control. CK, control check. Error bars indicate the SE of three biological repeats. (P < 0.05, Duncan’s multiple range test)

Salinity stress mainly influences Na^+^ and K^+^ distribution and homeostasis and high Na^+^ accumulation directly reduces carbon fixation and biomass production in plants (Munns and Tester 2008). To test if the *OsCESA9/OsCESA9*^*D387N*^ heterozygous genotype contributed to maintaining the ion balance in plants, we determined the content of Na^+^ and K^+^ in the shoots and roots of WT, *sdbc1* and F1 plants with and without salt treatment. There were lower Na^+^ levels in the shoots and roots of F1 plants under salt treatment, compared with the WT and *sdbc1* plants (Fig. 9a, b). The K^+^ content exhibited no significant differences in the shoots and roots of these plants under salt stress. Moreover, the K^+^/Na^+^ ratio in the shoots and roots of the F1 plants was higher than that of the WT and *sdbc1* plants under salt stress. Thus, the *OsCESA9/OsCESA9*^*D387N*^ heterozygous genotype affected K^+^/Na^+^ homeostasis and altered Na^+^ and K^+^ distribution between the shoots and roots. These resulted in the OsCESA9/OsCESA9^D387N^ heterozygous plants demonstrating greater salt tolerance.

To determine the reason for the enhanced salt tolerance in OsCESA9/OsCESA9^D387N^ heterozygous plants, we examined the expression of related genes in WT, *sdbc1* and F1 organs in the control and salt treatments by qRT-PCR. *OsSOS1* encodes a rice plasma membrane Na^+^/H^+^ exchanger protein and is induced by salt stress (Shi et al. 2002). As shown in Fig. 9c, the expression level of *OsSOS1* was significantly higher in the *sdbc1* and F1 plants than the WT under both conditions. *OsHKT1;5*, a major gene contributing to Na^+^ removal from the xylem and salt tolerance (Ren et al. 2005), was up-regulated in *sdbc1* and F1 plants under both conditions (Fig. 9c). These results are consistent with the decrease in Na^+^ content in shoots and roots of F1 plants. Although no obvious differences were observed in the shoots and roots of these plants under salt stress, the expression of *OsKAT1*, encoding the K1 channel protein (Obata et al. 2007), was up-regulated in F1 plants (Fig. 9c). Under control conditions, the expression of OsKAT1 was significantly up-regulated in *sdbc1* and F1 plants (Fig. 9c). This result is consistent with the higher K^+^ content in *sdbc1* and F1 plants without salt stress. Together, the expression of the above genes is consistent or in conflict with the expectation according to their functions reported previously. The diverse alterations in gene expression indicated that the examined genes are indirectly responsible for the increased salt tolerance in OsCESA9/OsCESA9^D387N^ heterozygous plants.

We also investigated *OsCESA9* expression of WT plants that treated by different NaCl concentration. The qRT-PCR assay revealed that the expression of *OsCESA9* in the shoots and roots was remarkably down-regulated with the increase in salt concentration (Figure S10). These results suggest an association between *OsCESA9* and salt stress. To determine whether salt stress affect integrity of the cell wall, SEM was performed to observe the leaf sheath cross-sections of WT, *sdbc1* and F1 plants before and after salt stress. The observations revealed that the thickness of sclerenchyma cell wall among all plants were obviously decreased after salt stress (Figure S11). These results suggest an association between cell wall biosynthesis and salt stress.

### The OsCESA9/OsCESA9^D387N^ Heterozygous Plants Are Suitable for Use in Straw Return to the Field

Brittle culm mutants have potential prospect for straw treatment due to their lower cellulose contents and culm easily broken during harvest (Ye et al. 2015). Given that the *OsCESA9/OsCESA9*^*D387N*^ heterozygous plants also show lower cellulose contents and easily broken culms without any morphological abnormalities, including lodging resistance (Fig. 1f-k and S12), we considered that they may be suitable for use in the return of straw to the field. To assess this possibility, we carried out field harvesting experiments. The results showed that the internodes of *OsCESA9/OsCESA9*^*D387N*^ heterozygous plants were easily crushed by a rice combine harvester and evenly distributed into the field (Figure S12b, d, f), compared to WT (Figure S13a, c, e). Together, the *OsCESA9/OsCESA9*^*D387N*^ heterozygous plants are suitable for use in the return of straw to the field.

## Discussion

### OsCESA9^D387N^ Causes a Dominant Negative Effect on Inferior Mechanical Strength

In rice, OsCESA4, OsCESA7 and OsCESA9 are required for secondary cell wall cellulose biosynthesis (Tanaka et al. 2003; Xiong et al. 2010). Mutations in any of these *CESA* genes lead to a brittle culm phenotype and a reduction in cellulose content (Song et al. 2013; Wang et al. 2016a; Zhang et al. 2009). In this study, we isolated a novel *OsCESA9* allele, *sdbc1*, with a brittle culm and lower cellulose content. To date, many *oscesa9* mutants have been identified (Fig. 3c), but only the *Bc6* mutant caused by a missense mutation, R588G, shows a semi-dominant brittle phenotype. Here, *sdbc1* was caused by a missense mutation, D387N, in the first conserved aspartic acid residue of OsCESA9, which also resulted in the semi-dominant brittle phenotype. As the other OsCESA9 alleles are recessive, this suggests that the semi-dominant phenotypes of *Bc6* and *Sdbc1* are not caused by a dosage effect. We expressed the *sdbc1* mutant cDNA in WT plants resulted in a mild brittle culm phenotype (Fig. 3e). The introduction of the *bc6* mutant gene into WT plants previously caused decreased cellulose content and brittle phenotype (Kotake et al. 2011). Therefore, mutation of the two sites can interfere with the function of WT OsCESA9.

**Fig. 10:**
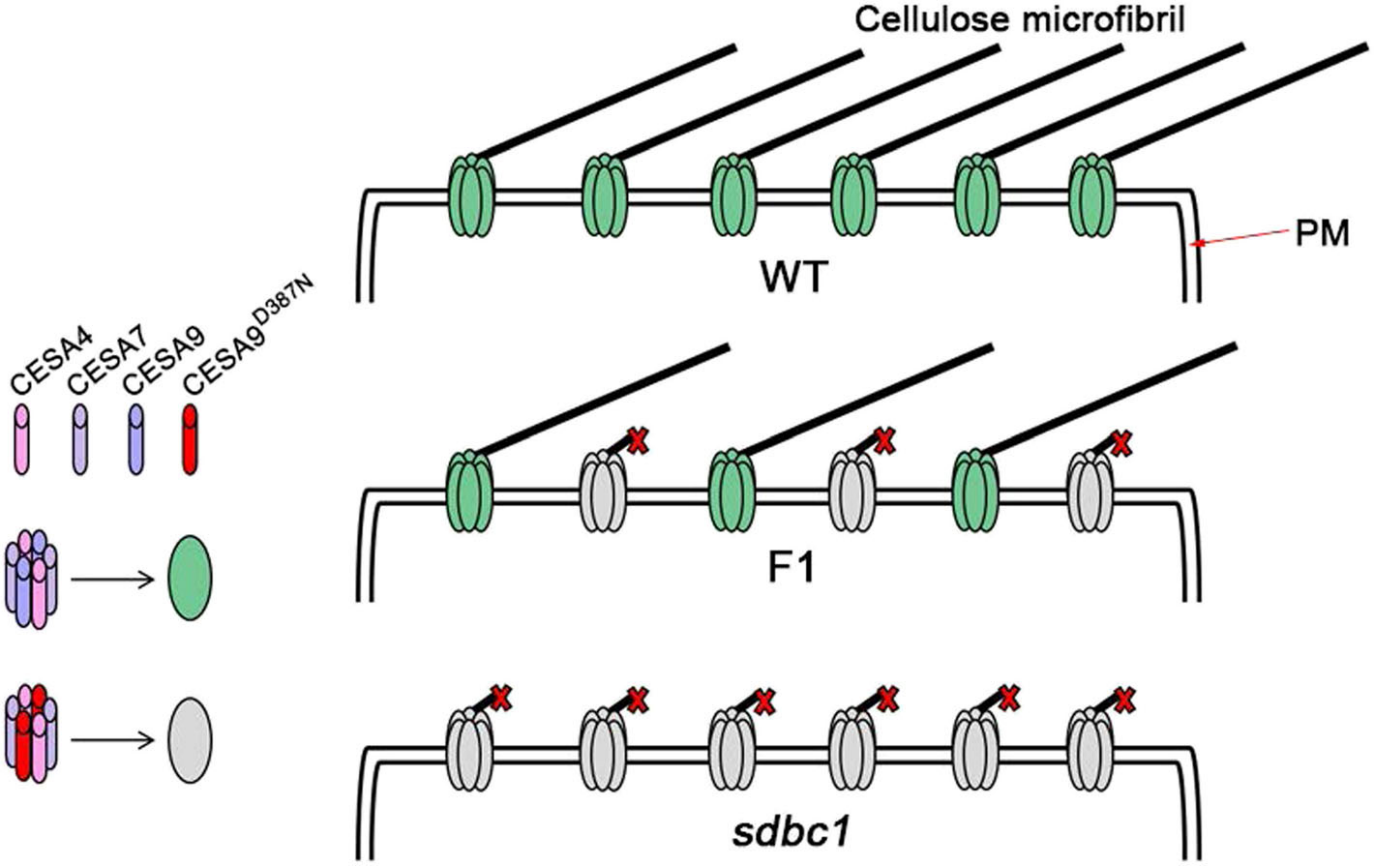
A hypothesis model of OsCESA9^D387N^ mutation. In the presence of OsCESA9^D387N^, OsCESA9^D387N^ can also interact with OsCESA4 and OsCESA9 to form non-functional or partially functional CSCs, and further affect cellulose synthesis. PM, plasma membrane

OsCESA9 shares common domains and motifs, such as zinc finger domains at the N-terminus, the D, D, D, QXXRW motif and eight TMDs, with all plant CESAs. Missense mutations located at distinct sites of OsCESA9 exhibit different phenotypes, indicating that these may play special roles in assigning functions to specific domains or motifs of the CESA proteins. The *sdbc1* mutation mapped to the first conserved aspartic acid residue, which is critical for substrate binding. We also verified that the *sdbc1* mutation does not affect its gene expression pattern, subcellular localization and interaction with OsCESA4 and OsCESA7. The OsCESA9^D387N^ mutation also does not affect the trafficking of secondary cell wall CSCs. Therefore, we speculated that the possible mechanism for the dominant negative effect of the *sdbc1* mutation is OsCESA9^D387N^ competing with the WT OsCESA9 for interaction with OsCESA4 and OsCESA7, and then forming nonfunctional or partially functional CSCs. In the *sdbc1* homozygous plants, only in the presence of OsCESA9^D387N^, the OsCESA9^D387N^ interacts with OsCESA4 and OsCESA7 for polymerizing nonfunctional or partially functional CESA4/7/9^D387N^ CSCs, affecting cellulose synthesis (Fig. 10). The normal CESA4/7/9 and abnormal CESA4/7/9^D387N^ CSCs simultaneously exist in the *Sdbc1* heterozygous plants (Fig. 10), so these exhibit the semi-dominant brittle phenotype. Unlike the *Bc6* mutation, OsCESA9^R588G^, which is located near to P586 corresponding to P557 of AtCESA7 that is altered in the semi-dominant *fra5* mutant of *Arabidopsis* (Kotake et al. 2011; Zhong et al. 2003). A study on the *Arabidopsis fra5* mutant suggested that the missense P557T mutation of AtCesA7 affects the interaction between CESA proteins or between CESA and other cellular components. Hence, *Bc6* may share the common mechanism for the dominant negative effect, with the *fra5* mutant (Kotake et al. 2011). Therefore, although the *Sdbc1* and *Bc6* mutants exhibited a semi-dominant brittle phenotype, the mechanism for the dominant negative effect differs. The *sdbc1* and *bc6* mutation occurs in the region between TMD2 and TMD3, but not all mutations that occur in this region exhibit semi-dominant phenotype, such as the *osfc16* and *bc88* mutations located between *sdbc1* and *bc6* mutation sites (Fig. 3c), However, these two mutants are recessive and show obviously different phenotypes (Li et al. 2017; Rao et al. 2013). The *osfc16* mutant with substitutions (W481C, P482S) in OsCESA9 shows slightly affected plant growth and significantly reduced cellulose crystallinity and thinner secondary cell walls (Li et al. 2017). The *bc88* mutant, which harbors the substitution (P421L) in OsCESA9 exhibits a diversity of pleiotropic phenotypes, including a brittle culm phenotype, dwarfism, withered leaf tips at the seedling stage, and an 18-d delay in heading date at the mature stage (Rao et al. 2013). Studies of these mutants suggest that the different conserved sites in the central cytoplasmic domain play a distinct role in cellulose synthesis. Therefore, more novel OsCESA9 alleles have been identified, which will provide insight into how CESAs function and what biological functions cellulose plays in plants.

### The OsCESA9^D387N^ Mutation Confers Salt Tolerance in Heterozygous Plants

The plant cell wall plays a critical role in development and in adaptation to abiotic and biotic stresses (Keegstra 2010). Soil salinity is a key abiotic stress affecting crop productivity worldwide (Munns and Gilliham 2015). In this study, we found that *Sdbc1* heterozygous plants showed greater tolerance to salt. Abiotic stresses can increase ROS production, which can damage DNA, proteins and carbohydrates, finally lead to cell death. ROS also cause lipid peroxidation, cell membrane damage and MDA production. In *Sdbc1* heterozygous plants, H_2_O_2_, one ROS was present in low levels under salt stress, as revealed by DAB staining. Consistently, MDA content and REC were also reduced in *Sdbc1* heterozygous plants, compared with WT and *sdbc1* plants. Limiting Na^+^ accumulation in the tissue is the one of most important approaches evolved by plants for tolerating salinity (Munns and Tester 2008). Here, the *Sdbc1* heterozygous plants show lower Na^+^ accumulation in the shoots and roots compared with that of the WT and *sdbc1* homozygous plants, which may be the main reason why the *Sdbc1* heterozygous plants have better salt tolerance. Although the up-regulated expression of *OsSOS1* for Na^+^ exclusion from the roots into the rhizosphere and *OsHTK1;5* for recirculation from the shoots to the roots can explain the low Na^+^ content in shoots and roots of *Sdbc1* heterozygous plants, the reason why higher Na^+^ accumulated in the *sdbc1* homozygous plants is still unclear. Previous studies indicate that higher levels of soluble sugar accumulation in plants can increase drought and osmotic tolerance (Cao et al. 2011). As a cellulose synthase mutant, both *sdbc1* homozygous and *Sdbc1* heterozygous plants show increased soluble sugar contents, but only *Sdbc1* plants exhibit salt tolerance. These may indicate that a proper reduction in cellulose content is also very important for salt tolerance. The severe reduction in cellulose content in the *sdbc1* homozygous plants possibly explains why they show higher level Na^+^ accumulation, although the expressions of *OsSOS1* and *OsHKT1;5* were also up-regulated.

Previous studies have identified many types of *cesa* mutants in response in salt, drought and other stresses. For instance, *Arabidopsis cesa8*^lew2–1^ (L802F) (Chen et al. 2005) and *cesa3*^ixr1–1^ (G998D) (Scheible et al. 2001) confer drought and isoxaben resistance to the mutant plants. Rice *bc13*, another *OsCESA9* allele, with a mutation at the 101st residue (G101K) in the N-terminal region conserved with acidic amino acids just after the zinc fingers, confers cadmium (Cd) tolerance and lower Cd accumulation in the grain (Song et al. 2013). In our research, we also found that the retention of some heavy metals, such as Cd, lead (Pb), copper (Cu) in the grain of *sdbc1* homozygous and *Sdbc1* heterozygous plants was lower than in the WT plants (Figure S14). Hence, the *sdbc1* mutant may also have heavy metal resistance, but these assumptions need further study. This mutation could be introduced into other rice varieties for breeding low heavy metal accumulating rice, especially for the hybrid rice, as the *Sdbc1* heterozygous plants are simultaneously slat tolerance and have better agronomic traits. We designed the CAPS marker for distinguishing the WT and *sdbc1* background, which can be applied for the identification of the mutation point for marker-assisted selection.

### The OsCESA9^D387N^ Mutation Has Potential Prospect for Residue Management

As one of the most important staple food crops, rice produces significant quantities of agronomic biomass residue every year. The processing of these residues has been a challenge due to the lignocellulose recalcitrance of the secondary cell wall (Alam et al. 2019). Straw burning is preferred by farmers, as it is economical and convenient, but it causes environmental problems. The high cellulose content of the cell wall of rice straw directly leads to slow decomposition (Tian et al. 1992). Brittle culm rice mutants have lower cellulose contents and finer breakage during threshing, which may result in faster residue decomposition (Cabiles et al. 2008; Johnson et al. 2006). Unfortunately, most of these mutants have concomitant phenotypes such as dwarfism, low fertility and withering of the leaf apex, which makes them inadequate for breeding. Our previous study revealed that *cef1* plants, which also show a brittle culm and low cellulose content without abnormal morphology, have potential use in residue management (Ye et al. 2015). In this study, the *sdbc1* homozygous and *Sdbc1* heterozygous plants exhibited brittle culms and lower cellulose contents, as well as no morphological abnormalities. Under field conditions, the lodging resistance and the grain yield of the *sdbc1* homozygous and *Sdbc1* heterozygous plants did not differ compared with the WT, but the *Sdbc1* heterozygous plants were easily crushed by the rice combine harvester and could be evenly distributed into the field. Therefore, the OsCESA9^D387N^ mutation improves the processing of rice straw for return to the field.

### The OsCESA9^D387N^ Mutation Displays Enhanced Biomass Enzymatic Saccharification

Plant cell wall represents the most abundant renewable biomass resource for biofuels on the earth. Three major steps are necessary for conversion of lignocellulose to ethanol. i) physical and chemical pretreatments to enhance cell wall destruction, ii) enzymatic digestion to release soluble sugars, and iii) microbial fermentation to produce ethanol (Wang et al. 2016b). The first two of the three steps are mainly affected by lignocellulose recalcitrance of the secondary cell wall. Cellulose is a principal component of plant cell walls and its content and features affect lignocellulose recalcitrance (Li et al. 2017; Li et al. 2018). In recent years, rice straw has been highlighted as an important material for biofuel production, but the high cellulose content and crystallinity determine lignocellulose recalcitrance, leading to costly biomass processing (Alam et al. 2019; Himmel et al. 2007). Here, we report that the *sdbc1* mutant has lower cellulose contents and culm easily broken during pretreatment. Biomass enzymatic saccharification efficiency is an important parameter for determining lignocellulosic straw digestibility. And we detected higher saccharification efficiency of the lignocellulosic material derived from the *sdbc1* homozygous and *Sdbc1* heterozygous plants, suggesting that the OsCESA9^D387N^ mutation can enhance biomass enzymatic saccharification (Fig. 4). Hybrid rice not only has higher grain yield, but it also produces greater biomass than that of conventional rice varieties (Jiang et al. 2016). Given that the OsCESA9^D387N^ mutation results in a dominant negative effect on cellulose biosynthesis, the OsCESA9^D387N^ mutation can be introduced into elite sterile lines or restorer lines to further resolve the straw processing issues of hybrid rice.

## Conclusions

In this study, we isolated a semi-dominant brittle culm (*sdbc*) phenotype. Map-based cloning revealed a substitution (D387N) at the first conserved aspartic acid residues of OsCESA9 corresponding to the mutant phenotypes. Further experiments indicate that mutation OsCESA9^D387N^ may compete with WT OsCESA9 for interaction with OsCESA4 and OsCESA7 and further to form non-functional or partially functional CSCs. The *sdbc1* homozygous and *Sdbc1* heterozygous plants show lower cellulose content and reduction in secondary wall thickness and enhances biomass enzymatic saccharification compared with WT. Not only that, we found that the OsCESA9/OsCESA9^D387N^ heterozygous plants increase salt tolerance through indirectly affecting related genes expression and improve straw returning to field due to its easily broken culms and lower cellulose content without any negative effects in plant normal growth and lodging. Hence, OsCESA9^D387N^ allele can improve rice salt tolerance and provide the prospect of the rice straw for biofuels and bioproducts due to its improved enzymatic saccharification.

## Supplementary Information

**Additional file 1.**

**Additional file 2: Table S1.** F1 phenotype and the number of plants with different segregation phenotype in F_2_ generation.

**Additional file 3: Figure S1**. (a) The seedling phenotype of WT, *sdbc1* and F1 plants. (b) Folding the seedling of WT, *sdbc1* and F1 plants. The *sdbc1* homozygous plants show brittle phenotype in seedling stage. Bars = 10 cm.

**Additional file 4: Figure S2.** Proportions of cell wall fractions. (a) Hemicellulose contents. (b) Pectin contents. (c) Starch contents. Error bars indicate SE from the mean of five replicates. Different letters denote significant differences (*P* < 0.05, Duncan’s multiple range test).

**Additional file 5: Figure S3.** Sequencing of WT, *sdbc1* and F1 plants, the red arrows indicate mutation site.

**Additional file 6: Figure S4.** Alignment of amino acid sequences of SDBC1 with other OsCESAs and AtCESAs. The red arrow indicates that the mutation site locates on the first conserved aspartic acid residues domain.

**Additional file 7: Figure S5.** The expression patterns of *OsCESA4* and *OsCESA7* in various rice organs of WT, *sdbc1* and F1 plants. The *Actin1* gene was used as an internal control.

**Additional file 8: Figure S6.** The OsCESA9^D387N^ mutation does not affect the interaction intensity with OsCESA4 and OsCESA7.

**Additional file 9: Figure S7.** Phenotypes of younger plants under treatment of 100 mM NaCl. Three-week-old plants were treated by 100 mM NaCl for ten days. Bar = 3 cm. (a) and (b) are the same plants with different angles for taking photo.

**Additional file 10: Figure S8.** Physiological analyses of WT, *sdbc1* and F1 plants under salt stress. Salt stress was performed with 200 mM NaCl for 96 h, whereas plants from the control group were maintained under hydroponic culture. (a) DAB staining of the first upper leaves of WT, *sdbc1* and F1 plants. Bars = 2 cm. (b) MDA contents of leaves from WT, *sdbc1* and F1plants following 200 mM NaCl treatment. (c) Relative electrical conductivity (REC) for WT, *sdbc1* and F1 plants. (d) Relative proline contents for WT, *sdbc1* and F1 plants. Error bars indicate the SE of three biological repeats. Different letters denote significant differences (P < 0.05, Duncan’s multiple range test). CK, control check; FW, fresh weight.

**Additional file 11: Figure S9.** The expression levels of genes that encode for Na^+^ and K^+^ transporters in WT, *sdbc1* and F1 plants. The *Actin1* gene was used as an internal control. CK, control check. Error bars indicate the SE of three biological repeats.

**Additional file 12: Figure S10.** Relative expression levels of *OsCESA9* in roots and shoots of WT with or without salt treatments. The *Actin1* gene was used as an internal control. Error bars indicate the SE of three biological repeats.

**Additional file 13: Figure S11.** Scanning electron micrographs of the sclerenchyma cell walls of WT, *sdbc1* and F1 plant with or without salt treatments. (b), (d), (f), (h), (j) and (l) are enlargements of the red boxed areas in (a), (c), (e), (g), (i) and (k) respectively. Bars = 20 μm (a, c, e, g, i, k) and 2 μm in (b, d, f, h, j, l). (m) The thickness of sclerenchyma cell walls. Error bars represent SE (*n* = 30). Different letters denote significant differences (P < 0.05, Duncan’s multiple range test). CK, control check.

**Additional file 14: Figure S12.** Lodging index of WT, *sdbc1* and F1 plants.

**Additional file 15: Figure S13.** The *Sdbc1* heterozygous plants easily smash by rice combine. (a) and (b) The WT and *Sdbc1* heterozygous plants are harvesting by rice combine, respectively. (c) and (d) The WT and *Sdbc1* heterozygous plants clums after harvest, respectively. (e) and (f) The length of WT and *Sdbc1* heterozygous plants culms distribution after harvest.

**Additional file 16: Figure S14.** The content of Cd, Pb and Cu in WT, *sdbc1* and F1 grains. Error bars indicate SE from the mean of three replicates. Different letters denote significant differences (P < 0.05, Duncan’s multiple range test).

## Data Availability

Not applicable.

## Abbreviations

CESA: cellulose synthase
CSC: cellulose synthase complex
TMD: transmembrane domain
BC: brittle culm
DAB: 3,3′-Diaminobenzidine
REC: relative electrical conductivity
SFLC: split firefly luciferase complementation

## References

[CR1] Alam A, Zhang R, Liu P, Huang J, Wang Y, Hu Z, Madadi M, Sun D, Hu R, Ragauskas AJ (2019). A finalized determinant for complete lignocellulose enzymatic saccharification potential to maximize bioethanol production in bioenergy Miscanthus. Biotechnol Biofuels.

[CR2] Cabiles DMS, Angeles OR, Johnson-Beebout SE, Sanchez PB, Buresh RJ (2008). Faster residue decomposition of brittle stem rice mutant due to finer breakage during threshing. Soil Tillage Res.

[CR3] Cao H, Guo S, Xu Y, Jiang K, Jones AM, Chong K (2011). Reduced expression of a gene encoding a Golgi localized monosaccharide transporter (OsGMST1) confers hypersensitivity to salt in rice (Oryza sativa). J Exp Bot.

[CR4] Cao WH, Liu J, He XJ, Mu RL, Zhou HL, Chen SY, Zhang JS (2007). Modulation of ethylene responses affects plant salt-stress responses. Plant Physiol.

[CR5] Chen H, Zou Y, Shang Y, Lin H, Wang Y, Cai R, Tang X, Zhou JM (2008). Firefly luciferase complementation imaging assay for protein-protein interactions in plants. Plant Physiol.

[CR6] Chen Z, Hong X, Zhang H, Wang Y, Li X, Zhu JK, Gong Z (2005). Disruption of the cellulose synthase gene, AtCesA8/IRX1, enhances drought and osmotic stress tolerance in Arabidopsis. Plant J.

[CR7] Correa-Ferreira ML, Viudes EB, de Magalhaes PM, Paixao de Santana Filho A, Sassaki GL, Pacheco AC, de Oliveira Petkowicz CL (2019). Changes in the composition and structure of cell wall polysaccharides from Artemisia annua in response to salt stress. Carbohydr Res.

[CR8] Fang, C, Li, K, Wu, Y, Wang, D, Zhou, J, Liu, X, Li, Y, Jin, C, Liu, X, Mur, LAJ, et al. (2019). OsTSD2-mediated cell wall modification affects ion homeostasis and salt tolerance. Plant Cell Environ 42:1503-1512. 10.1111/pce.13499 30536744

[CR9] Gao Y, He C, Zhang D, Liu X, Xu Z, Tian Y, Liu XH, Zang S, Pauly M, Zhou Y, Zhang B (2017). Two Trichome birefringence-like proteins mediate Xylan acetylation, which is essential for leaf blight resistance in Rice. Plant Physiol.

[CR10] Himmel ME, Ding SY, Johnson DK, Adney WS, Nimlos MR, Brady JW, Foust TD (2007). Biomass recalcitrance: engineering plants and enzymes for biofuels production. Science.

[CR11] Hofte H, Voxeur A (2017). Plant cell walls. Curr Biol.

[CR12] Jiang P, Xie X, Huang M, Zhou X, Zhang R, Chen J, Wu D, Xia B, Xiong H, Xu F, Zou Y (2016). Potential yield increase of hybrid Rice at five locations in southern China. Rice (N Y).

[CR13] Johnson SE, Angeles OR, Brar DS, Buresh RJ (2006). Faster anaerobic decomposition of a brittle straw rice mutant: implications for residue management. Soil Biol Biochem.

[CR14] Joshi CP, Mansfield SD (2007). The cellulose paradox--simple molecule, complex biosynthesis. Curr Opin Plant Biol.

[CR15] Keegstra K (2010). Plant cell walls. Plant Physiol.

[CR16] Kotake T, Aohara T, Hirano K, Sato A, Kaneko Y, Tsumuraya Y, Takatsuji H, Kawasaki S (2011). Rice brittle culm 6 encodes a dominant-negative form of CesA protein that perturbs cellulose synthesis in secondary cell walls. J Exp Bot.

[CR17] Li F, Liu S, Xu H, Xu Q (2018). A novel FC17/CESA4 mutation causes increased biomass saccharification and lodging resistance by remodeling cell wall in rice. Biotechnol Biofuels.

[CR18] Li F, Xie G, Huang J, Zhang R, Li Y, Zhang M, Wang Y, Li A, Li X, Xia T, Qu C, Hu F, Ragauskas AJ, Peng L (2017). OsCESA9 conserved-site mutation leads to largely enhanced plant lodging resistance and biomass enzymatic saccharification by reducing cellulose DP and crystallinity in rice. Plant Biotechnol J.

[CR19] Li J, Wang X, Wang X, Ma P, Yin W, Wang Y, Chen Y, Chen S, Jia H (2020). Hydrogen sulfide promotes hypocotyl elongation via increasing cellulose content and changing the arrangement of cellulose fibrils in alfalfa. J Exp Bot.

[CR20] McFarlane HE, Doring A, Persson S (2014). The cell biology of cellulose synthesis. Annu Rev Plant Biol.

[CR21] Munns R, Gilliham M (2015). Salinity tolerance of crops - what is the cost?. New Phytol.

[CR22] Munns R, Tester M (2008). Mechanisms of salinity tolerance. Annu Rev Plant Biol.

[CR23] Obata T, Kitamoto HK, Nakamura A, Fukuda A, Tanaka Y (2007). Rice shaker potassium channel OsKAT1 confers tolerance to salinity stress on yeast and rice cells. Plant Physiol.

[CR24] Obrdlik P, El-Bakkoury M, Hamacher T, Cappellaro C, Vilarino C, Fleischer C, Ellerbrok H, Kamuzinzi R, Ledent V, Blaudez D, Sanders D, Revuelta JL, Boles E, Andre B, Frommer WB (2004). K+ channel interactions detected by a genetic system optimized for systematic studies of membrane protein interactions. Proc Natl Acad Sci U S A.

[CR25] Ouyang SQ, Liu YF, Liu P, Lei G, He SJ, Ma B, Zhang WK, Zhang JS, Chen SY (2010). Receptor-like kinase OsSIK1 improves drought and salt stress tolerance in rice (Oryza sativa) plants. Plant J.

[CR26] Palmeros-Suarez PA, Massange-Sanchez JA, Sanchez-Segura L, Martinez-Gallardo NA, Espitia Rangel E, Gomez-Leyva JF, Delano-Frier JP (2017). AhDGR2, an amaranth abiotic stress-induced DUF642 protein gene, modifies cell wall structure and composition and causes salt and ABA hyper-sensibility in transgenic Arabidopsis. Planta.

[CR27] Pear JR, Kawagoe Y, Schreckengost WE, Delmer DP, Stalker DM (1996). Higher plants contain homologs of the bacterial celA genes encoding the catalytic subunit of cellulose synthase. Proc Natl Acad Sci U S A.

[CR28] Rao Y, Yang Y, Xin D, Li X, Zhai K, Ma B, Pan J, Qian Q, Zeng D (2013). Characterization and cloning of a brittle culm mutant (bc88) in rice (*Oryza sativa L.*). Chin Sci Bull.

[CR29] Ren ZH, Gao JP, Li LG, Cai XL, Huang W, Chao DY, Zhu MZ, Wang ZY, Luan S, Lin HX (2005). A rice quantitative trait locus for salt tolerance encodes a sodium transporter. Nat Genet.

[CR30] Sathitsuksanoh N, Xu B, Zhao B, Zhang YH (2013). Overcoming biomass recalcitrance by combining genetically modified switchgrass and cellulose solvent-based lignocellulose pretreatment. PLoS One.

[CR31] Scheible WR, Eshed R, Richmond T, Delmer D, Somerville C (2001). Modifications of cellulose synthase confer resistance to isoxaben and thiazolidinone herbicides in Arabidopsis Ixr1 mutants. Proc Natl Acad Sci U S A.

[CR32] Shi H, Quintero FJ, Pardo JM, Zhu JK (2002). The putative plasma membrane Na(+)/H(+) antiporter SOS1 controls long-distance Na(+) transport in plants. Plant Cell.

[CR33] Somerville C (2006). Cellulose synthesis in higher plants. Annu Rev Cell Dev Biol.

[CR34] Song XQ, Liu LF, Jiang YJ, Zhang BC, Gao YP, Liu XL, Lin QS, Ling HQ, Zhou YH (2013). Disruption of secondary wall cellulose biosynthesis alters cadmium translocation and tolerance in rice plants. Mol Plant.

[CR35] Tanaka K, Murata K, Yamazaki M, Onosato K, Miyao A, Hirochika H (2003). Three distinct rice cellulose synthase catalytic subunit genes required for cellulose synthesis in the secondary wall. Plant Physiol.

[CR36] Taylor NG, Howells RM, Huttly AK, Vickers K, Turner SR (2003). Interactions among three distinct CesA proteins essential for cellulose synthesis. Proc Natl Acad Sci U S A.

[CR37] Tian G, Kang B, Brussaard L (1992). Biological effects of plant residues with contrasting chemical compositions under humid tropical conditions—decomposition and nutrient release. Soil Biol Biochem.

[CR38] Updegraff DM (1969). Semimicro determination of cellulose in biological materials. Anal Biochem.

[CR39] Vermerris W, Abril A (2015). Enhancing cellulose utilization for fuels and chemicals by genetic modification of plant cell wall architecture. Curr Opin Biotechnol.

[CR40] Wadsworth GJ, Redinbaugh MG, Scandalios JG (1988). A procedure for the small-scale isolation of plant RNA suitable for RNA blot analysis. Anal Biochem.

[CR41] Wang D, Qin Y, Fang J, Yuan S, Peng L, Zhao J, Li X (2016). A missense mutation in the zinc finger domain of OsCESA7 deleteriously affects cellulose biosynthesis and plant growth in Rice. PLoS One.

[CR42] Wang D, Yuan S, Yin L, Zhao J, Guo B, Lan J, Li X (2012). A missense mutation in the transmembrane domain of CESA9 affects cell wall biosynthesis and plant growth in rice. Plant Sci.

[CR43] Wang Y, Fan C, Hu H, Li Y, Sun D, Wang Y, Peng L (2016). Genetic modification of plant cell walls to enhance biomass yield and biofuel production in bioenergy crops. Biotechnol Adv.

[CR44] Wong ML, Medrano JF (2005). Real-time PCR for mRNA quantitation. Biotechniques.

[CR45] Xiong G, Li R, Qian Q, Song X, Liu X, Yu Y, Zeng D, Wan J, Li J, Zhou Y (2010). The rice dynamin-related protein DRP2B mediates membrane trafficking, and thereby plays a critical role in secondary cell wall cellulose biosynthesis. Plant J.

[CR46] Ye Y, Liu B, Zhao M, Wu K, Cheng W, Chen X, Liu Q, Liu Z, Fu X, Wu Y (2015). CEF1/OsMYB103L is involved in GA-mediated regulation of secondary wall biosynthesis in rice. Plant Mol Biol.

[CR47] Ye Y, Wu K, Chen J, Liu Q, Wu Y, Liu B, Fu X (2018). OsSND2, a NAC family transcription factor, is involved in secondary cell wall biosynthesis through regulating MYBs expression in rice. Rice (N Y).

[CR48] Zhang B, Deng L, Qian Q, Xiong G, Zeng D, Li R, Guo L, Li J, Zhou Y (2009). A missense mutation in the transmembrane domain of CESA4 affects protein abundance in the plasma membrane and results in abnormal cell wall biosynthesis in rice. Plant Mol Biol.

[CR49] Zhong R, Morrison WH, Freshour GD, Hahn MG, Ye ZH (2003). Expression of a mutant form of cellulose synthase AtCesA7 causes dominant negative effect on cellulose biosynthesis. Plant Physiol.

[CR50] Zhou Y, Li S, Qian Q, Zeng D, Zhang M, Guo L, Liu X, Zhang B, Deng L, Liu X, Luo G, Wang X, Li J (2009). BC10, a DUF266-containing and Golgi-located type II membrane protein, is required for cell-wall biosynthesis in rice (*Oryza sativa L.*). Plant J.

