# Supplementary material for: A Semi-Dominant Mutation in OsCESA9 Improves Salt Tolerance and Favors Field Straw Decay Traits by Altering Cell Wall Properties in Rice
Source: Rice (N Y). 2021 Feb 17;14:19. doi: 10.1186/s12284-021-00457-0 (PMC7889784; doi:10.1186/s12284-021-00457-0)
Supplement: Supplementary file 2 — Additional file 2: Table S1. F1 phenotype and the number of plants with different segregation phenotype in F2 generation. [file 12284_2021_457_MOESM2_ESM.docx]

| cross combinations | F_1_ phenotype | F_2_ generation | | |
| --- | --- | --- | --- | --- |
|  |  | Normal (1) | Mild brittle (2) | Easily brittle (1) |
| WT/*sdbc1* | Mild brittle | 35 | 72 | 32 |
| XS110/*sdbc1* | Mild brittle | 46 | 91 | 43 |
| 93-11/*sdbc1* | Mild brittle | 37 | 75 | 33 |

Table S1. F1 phenotype and the number of plants with different segregation phenotype in F_2_ generation
