# Supplementary figures and images for: A Semi-Dominant Mutation in OsCESA9 Improves Salt Tolerance and Favors Field Straw Decay Traits by Altering Cell Wall Properties in Rice
Source: Rice (N Y). 2021 Feb 17;14:19. doi: 10.1186/s12284-021-00457-0 (PMC7889784; doi:10.1186/s12284-021-00457-0)

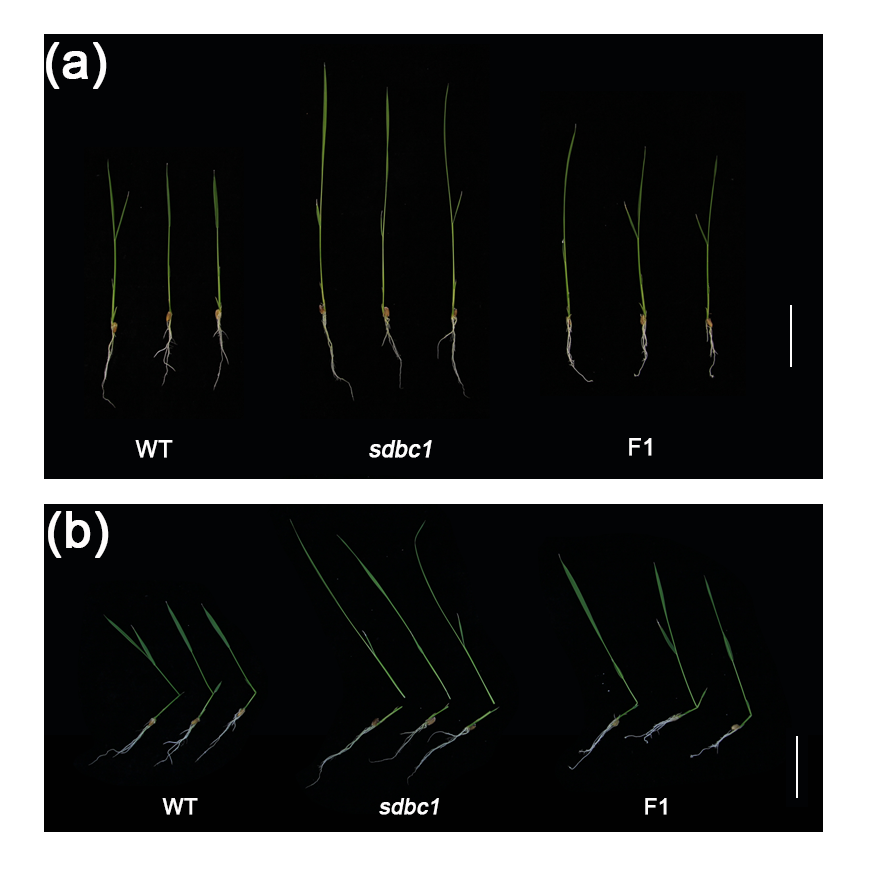

Supplement: Supplementary file 3 — Additional file 3: Figure S1. (a) The seedling phenotype of WT, sdbc1 and F1 plants. (b) Folding the seedling of WT, sdbc1 and F1 plants. The sdbc1 homozygous plants show brittle phenotype in seedling stage. Bars = 10 cm. [file 12284_2021_457_MOESM3_ESM.tif]

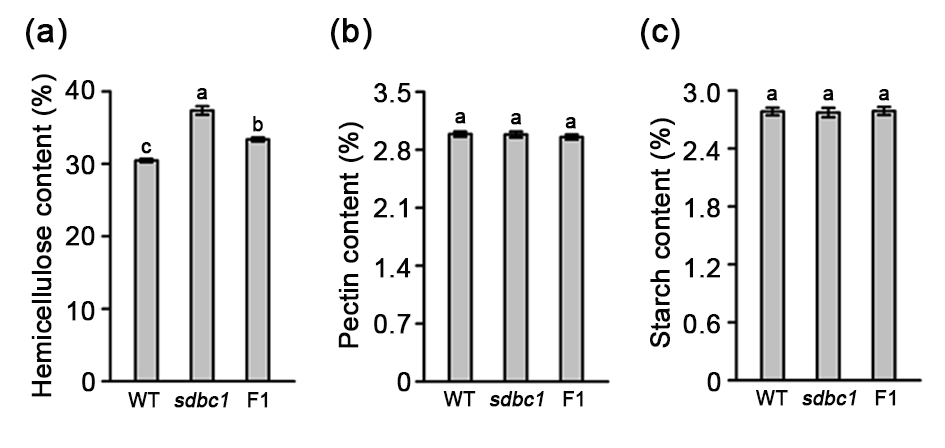

Supplement: Supplementary file 4 — Additional file 4: Figure S2. Proportions of cell wall fractions. (a) Hemicellulose contents. (b) Pectin contents. (c) Starch contents. Error bars indicate SE from the mean of five replicates. Different letters denote significant differences (P < 0.05, Duncan’s multiple range test). [file 12284_2021_457_MOESM4_ESM.tif]

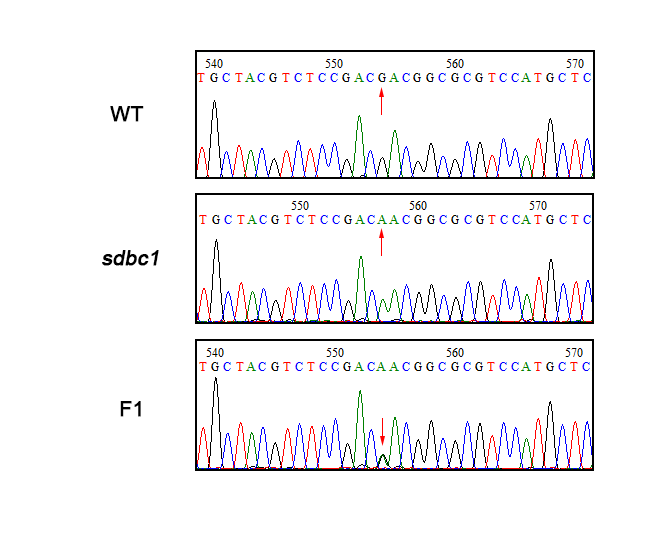

Supplement: Supplementary file 5 — Additional file 5: Figure S3. Sequencing of WT, sdbc1 and F1 plants, the red arrows indicate mutation site. [file 12284_2021_457_MOESM5_ESM.tif]

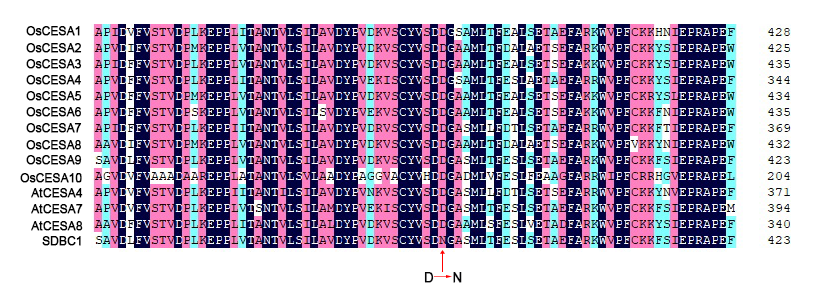

Supplement: Supplementary file 6 — Additional file 6: Figure S4. Alignment of amino acid sequences of SDBC1 with other OsCESAs and AtCESAs. The red arrow indicates that the mutation site locates on the first conserved aspartic acid residues domain. [file 12284_2021_457_MOESM6_ESM.tif]

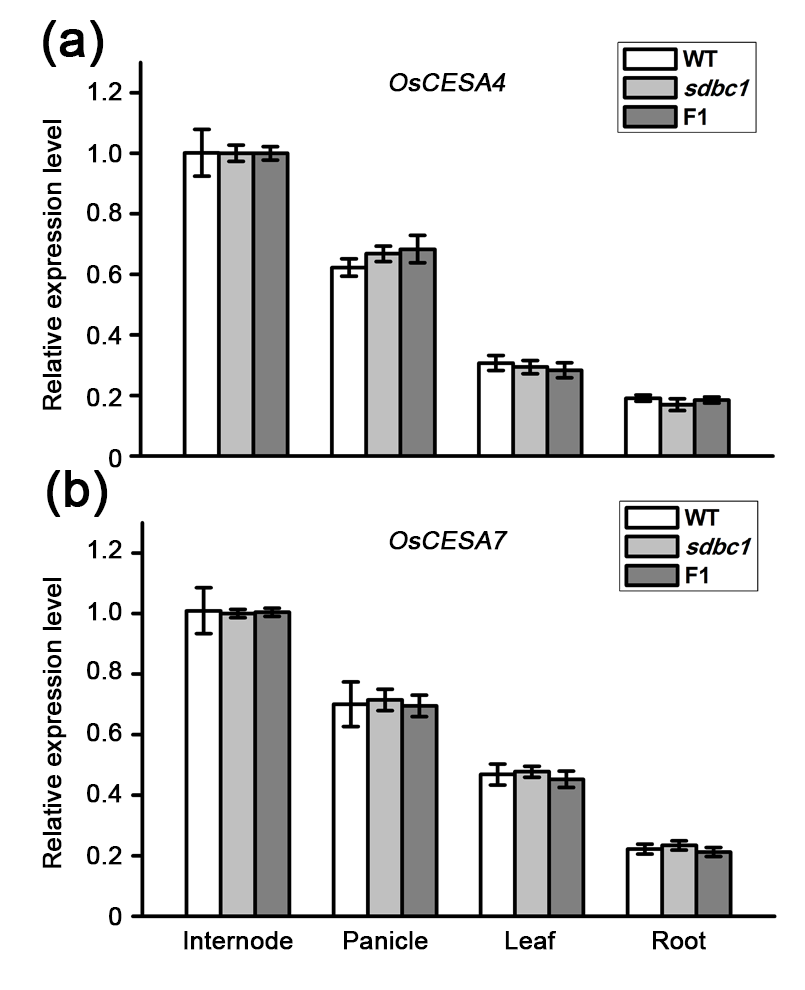

Supplement: Supplementary file 7 — Additional file 7: Figure S5. The expression patterns of OsCESA4 and OsCESA7 in various rice organs of WT, sdbc1 and F1 plants. The Actin1 gene was used as an internal control. [file 12284_2021_457_MOESM7_ESM.tif]

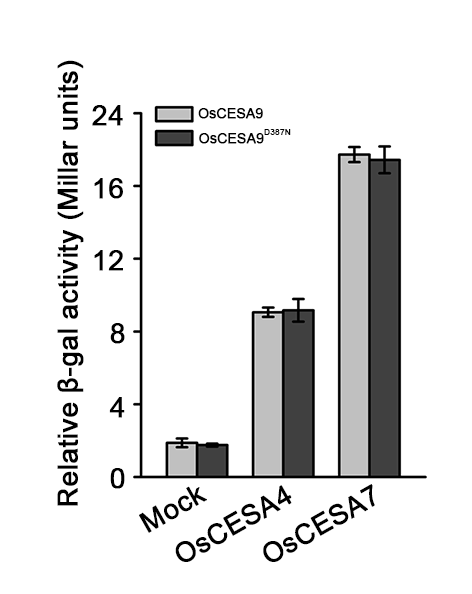

Supplement: Supplementary file 8 — Additional file 8: Figure S6. The OsCESA9D387N mutation does not affect the interaction intensity with OsCESA4 and OsCESA7. [file 12284_2021_457_MOESM8_ESM.tif]

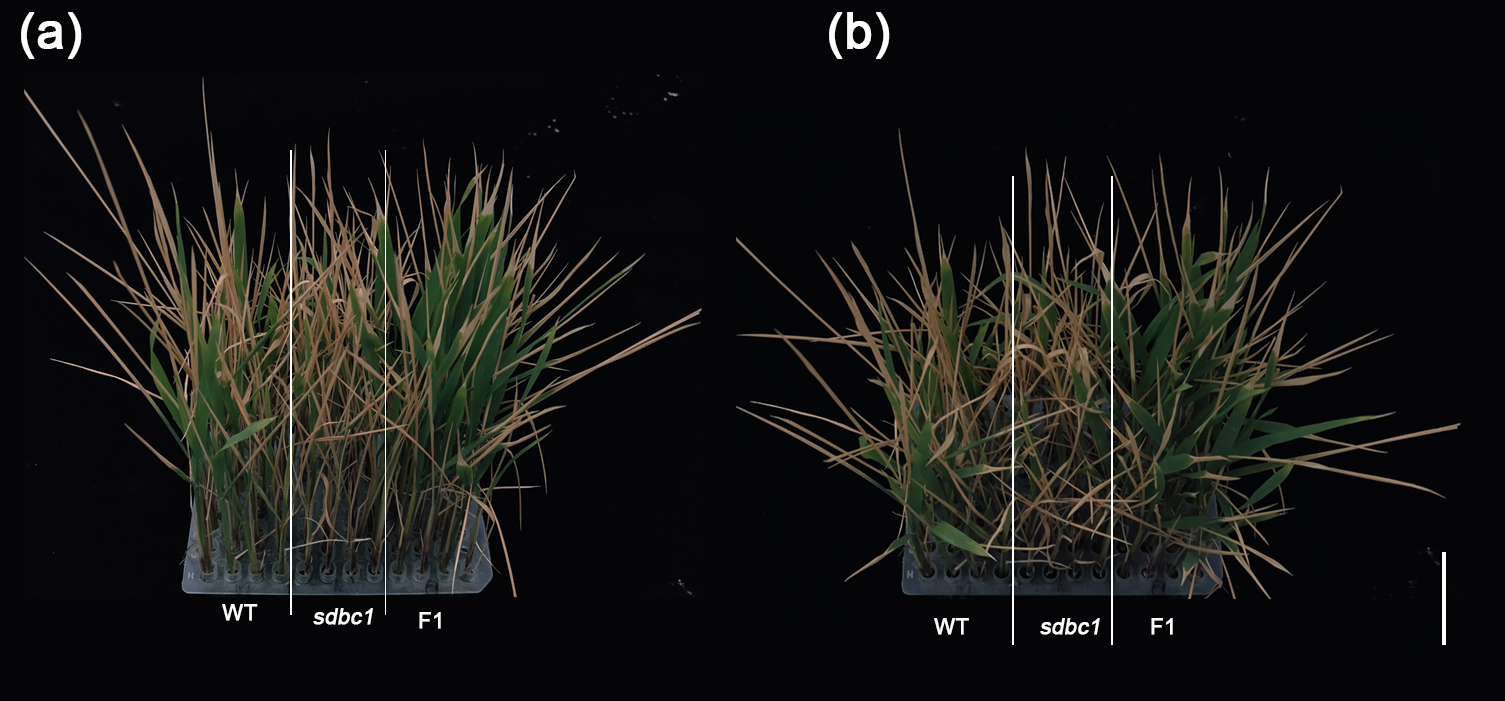

Supplement: Supplementary file 9 — Additional file 9: Figure S7. Phenotypes of younger plants under treatment of 100 mM NaCl. Three-week-old plants were treated by 100 mM NaCl for ten days. Bar = 3 cm. (a) and (b) are the same plants with different angles for taking photo. [file 12284_2021_457_MOESM9_ESM.tif]

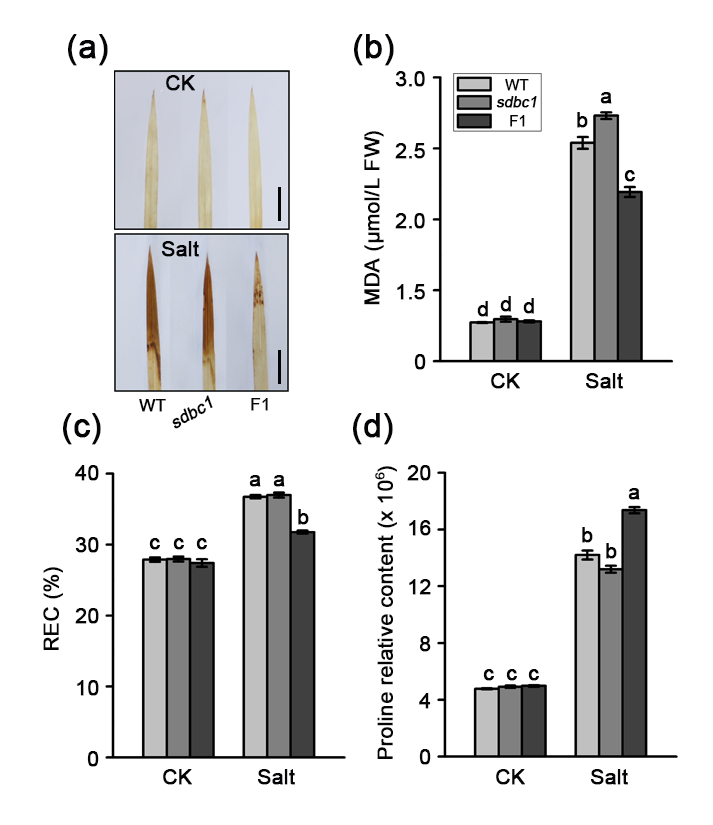

Supplement: Supplementary file 10 — Additional file 10: Figure S8. Physiological analyses of WT, sdbc1 and F1 plants under salt stress. Salt stress was performed with 200 mM NaCl for 96 h, whereas plants from the control group were maintained under hydroponic culture. (a) DAB staining of the first upper leaves of WT, sdbc1 and F1 plants. Bars = 2 cm. (b) MDA contents of leaves from WT, sdbc1 and F1plants following 200 mM NaCl treatment. (c) Relative electrical conductivity (REC) for WT, sdbc1 and F1 plants. (d) Relative proline contents for WT, sdbc1 and F1 plants. Error bars indicate the SE of three biological repeats. Different letters denote significant differences (P < 0.05, Duncan’s multiple range test). CK, control check; FW, fresh weight. [file 12284_2021_457_MOESM10_ESM.tif]

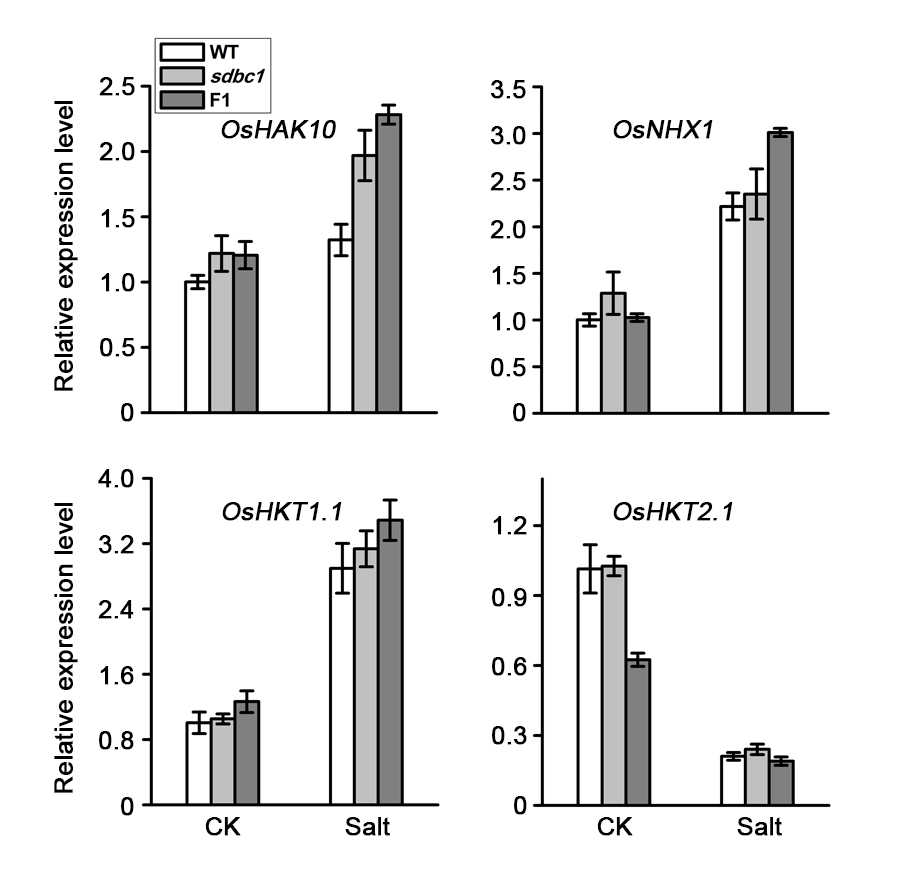

Supplement: Supplementary file 11 — Additional file 11: Figure S9. The expression levels of genes that encode for Na+ and K+ transporters in WT, sdbc1 and F1 plants. The Actin1 gene was used as an internal control. CK, control check. Error bars indicate the SE of three biological repeats. [file 12284_2021_457_MOESM11_ESM.tif]

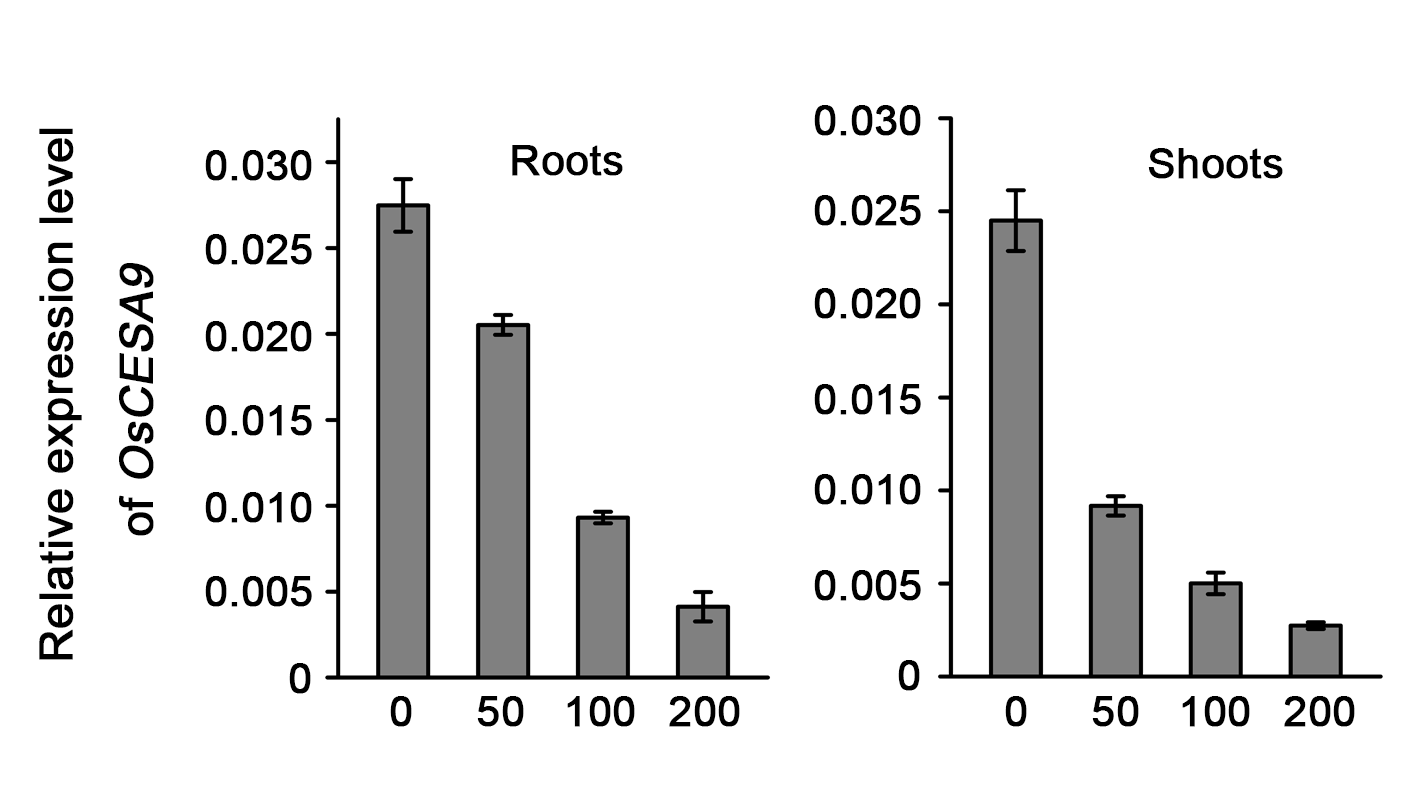

Supplement: Supplementary file 12 — Additional file 12: Figure S10. Relative expression levels of OsCESA9 in roots and shoots of WT with or without salt treatments. The Actin1 gene was used as an internal control. Error bars indicate the SE of three biological repeats. [file 12284_2021_457_MOESM12_ESM.tif]

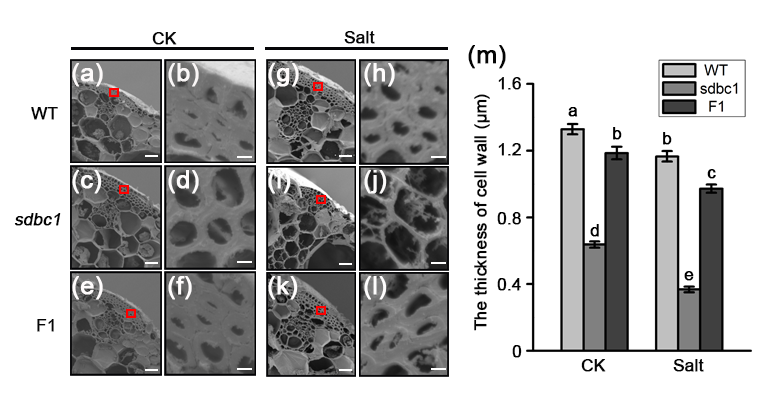

Supplement: Supplementary file 13 — Additional file 13: Figure S11. Scanning electron micrographs of the sclerenchyma cell walls of WT, sdbc1 and F1 plant with or without salt treatments. (b), (d), (f), (h), (j) and (l) are enlargements of the red boxed areas in (a), (c), (e), (g), (i) and (k) respectively. Bars = 20 μm (a, c, e, g, i, k) and 2 μm in (b, d, f, h, j, l). (m) The thickness of sclerenchyma cell walls. Error bars represent SE (n = 30). Different letters denote significant differences (P < 0.05, Duncan’s multiple range test). CK, control check. [file 12284_2021_457_MOESM13_ESM.tif]

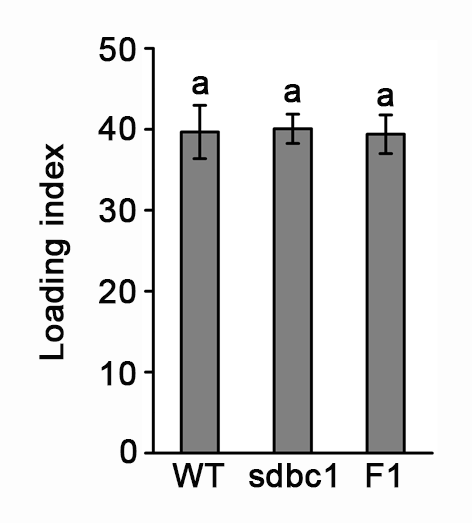

Supplement: Supplementary file 14 — Additional file 14: Figure S12. Lodging index of WT, sdbc1 and F1 plants. [file 12284_2021_457_MOESM14_ESM.tif]

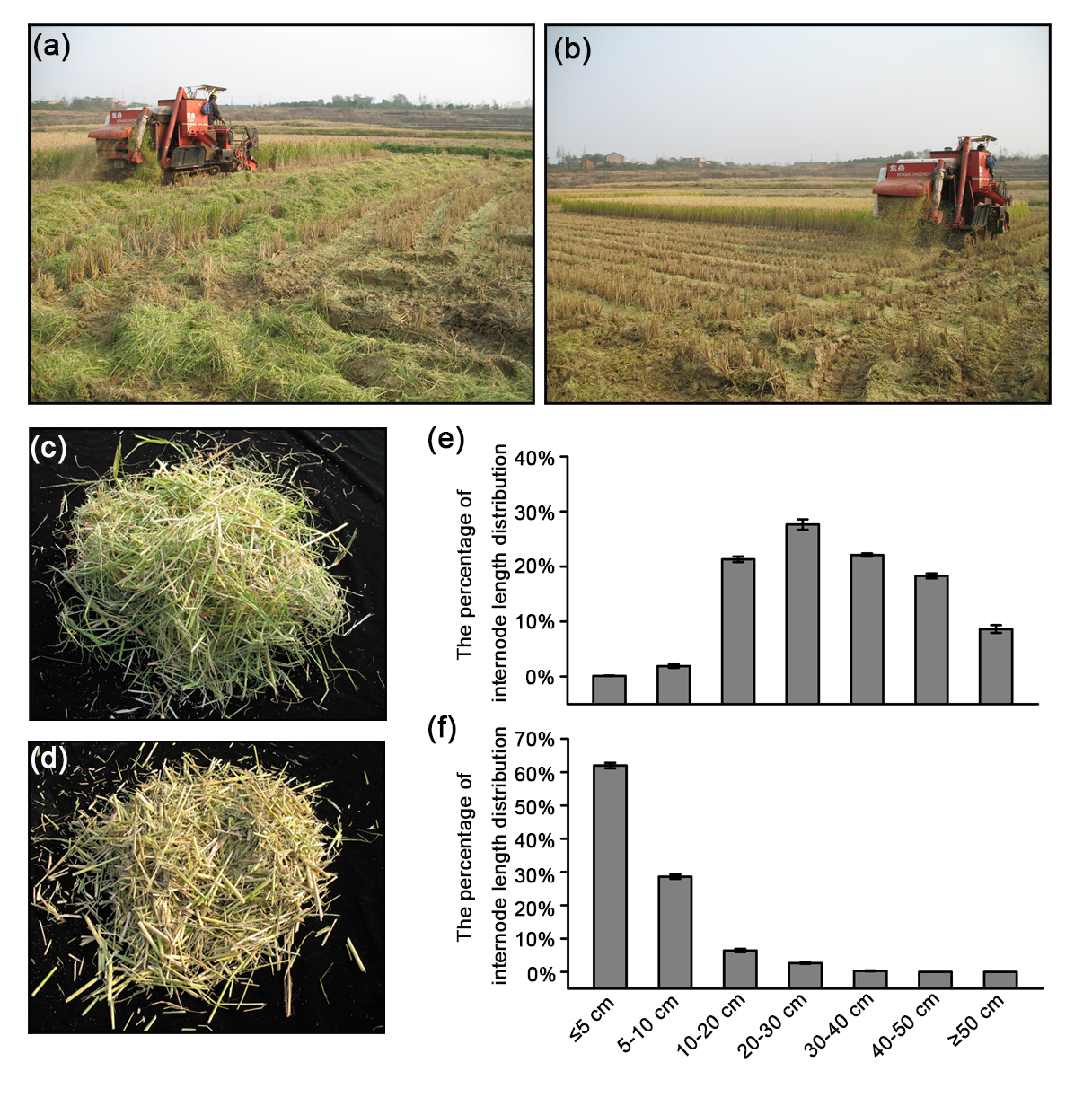

Supplement: Supplementary file 15 — Additional file 15: Figure S13. The Sdbc1 heterozygous plants easily smash by rice combine. (a) and (b) The WT and Sdbc1 heterozygous plants are harvesting by rice combine, respectively. (c) and (d) The WT and Sdbc1 heterozygous plants clums after harvest, respectively. (e) and (f) The length of WT and Sdbc1 heterozygous plants culms distribution after harvest. [file 12284_2021_457_MOESM15_ESM.tif]

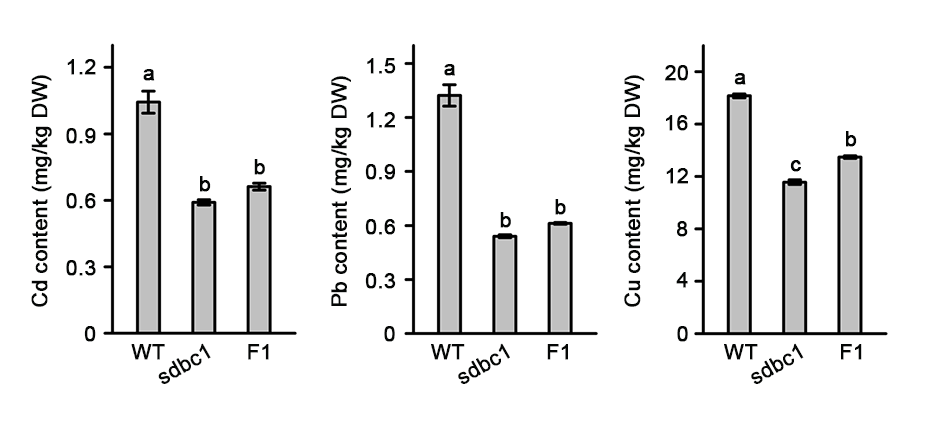

Supplement: Supplementary file 16 — Additional file 16: Figure S14. The content of Cd, Pb and Cu in WT, sdbc1 and F1 grains. Error bars indicate SE from the mean of three replicates. Different letters denote significant differences (P < 0.05, Duncan’s multiple range test). [file 12284_2021_457_MOESM16_ESM.tif]
